# METTL3/IGF2BP1 Axis Orchestrates m6A-Dependent NCAM1 Preservation to Combat Age-Related Cognitive Decline

**DOI:** 10.3390/cells15181662

**Published:** 2026-09-15

**Authors:** Guohua Ji, Yujie Zhao, Xu Liu, Xiaopeng Li, Liang Lu, Fengji Liang, Yanhong Yuan, Yuying Dai, Bo Li, Yanxiang Qu, Bo Song, Lina Qu

**Affiliations:** 1 State Key Laboratory of Space Medicine, China Astronaut Research and Training Center, Beijing 100094, China; 2 Department of Pathology, School of Basic Medical Sciences, Peking University Health Science Center, Beijing 100191, China; 3 Department of Pathology and Forensics, Dalian Medical University, Dalian 116044, China

**Keywords:** NCAM1, m6A, METTL3, senescence, learning and memory

## Abstract

**Highlights:**

**What are the main findings?**

- Hippocampal Ncam1 exhibits reduced m6A methylation and down-regulated expression in aged SAMP8 mice.
- METTL3-mediated m6A modification stabilizes Ncam1 mRNA via the reader protein IGF2BP1.
- Overexpression of METTL3/IGF2BP1-NCAM1 axis alleviates neuronal senescence and rescues age-related learning-memory deficits.

**What is the implication of the main finding?**

- This work defines a new epitranscriptomic regulatory axis for hippocampal aging and cognitive impairment.
- It links m6A RNA modification to synaptic plasticity maintenance in the aging brain.
- METTL3-IGF2BP1-NCAM1 may serve as potential targets for treating age-associated cognitive decline.

**Abstract:**

Age-related decline in learning and memory functions poses significant challenges in an aging society, with epigenetic dysregulation emerging as a key contributor to cognitive deterioration. As the most prevalent internal RNA modification, N6-methyladenosine (m6A) dynamically orchestrates neural transcriptome plasticity through its “writers,” “erasers,” and “readers,” yet its role in aging-associated cognitive impairment remains underexplored. This study employs an integrated epitranscriptomic approach to investigate m6A-mediated regulation in hippocampal aging processes. Through comparative m6A-mRNA epitranscriptomic microarray analysis of senescence-accelerated mouse prone 8 (SAMP8) and senescence-resistant SAMR1 hippocampi, we identified neural cell adhesion molecule 1 (NCAM1) as a key m6A-regulated effector whose decreased expression correlates with accelerated cognitive deterioration. Mechanistically, we revealed that Methyltransferase-like 3 (METTL3)-mediated m6A modification governs *Ncam1* mRNA stability through insulin-like growth factor 2 mRNA-binding protein 1 (IGF2BP1) reader protein-dependent mechanisms, forming a regulatory axis that modulates cyclic AMP response element-binding protein (CREB) signaling pathway activity. Remarkably, targeting of this METTL3/IGF2BP1/NCAM1 axis significantly attenuated cognitive deficits in aged SAMP8 mice. Our findings establish an m6A methylation-dependent paradigm for NCAM1-mediated cognitive preservation during aging, uncovering a novel epitranscriptomic layer in age-related neurodegeneration.

## 1. Introduction

Cognitive function, the cornerstone of human experience, encompasses the complex mental processes that govern perception, learning, memory, and decision-making [1,2]. These higher-order brain functions are particularly vulnerable to the progressive effects of aging, with cognitive decline emerging as a hallmark of the aging process [3]. Aging is associated with a progressive decline in cognitive function, particularly in learning and memory processes, posing significant challenges to healthy aging and quality of life [4,5]. Age-related cognitive impairment not only diminishes quality of life but also significantly increases susceptibility to neurodegenerative disorders such as Alzheimer’s disease (AD) in serious cases [6]. The intricate interplay between aging and cognitive function represents a critical area of scientific inquiry, offering insights into the molecular and cellular mechanisms underlying the natural decline of mental faculties [7].

While the molecular mechanisms underlying age-related cognitive decline remain incompletely understood, emerging evidence highlights the critical role of synaptic plasticity and its regulation by RNA modifications in maintaining cognitive health [8,9]. Among these modifications, N6-methyladenosine (m6A), the most abundant internal mRNA modification in eukaryotic cells, has recently emerged as a key regulator of neuronal function and aging [9,10]. The m6A modification is dynamically regulated by a complex interplay of “writers” (methyltransferases), “erasers” (demethylases), and “readers” (m6A-binding proteins) [11,12]. METTL3, the core catalytic subunit of the m6A methyltransferase complex, has been implicated in various aspects of neuronal development and function [13,14]. METTL3 has been shown to enhance long-term memory consolidation, likely by promoting the translation efficacy of activity-induced immediate early genes (IEGs) [15]. Similarly, IGF2BP1, an m6A reader protein, plays crucial roles in mRNA stability and translation, particularly in neural tissues [16,17]. Mice with genetic deletion of the m6A reader YTHDF1 exhibit learning and memory defects, along with impaired hippocampal synaptic transmission and long-term potentiation [18]. Furthermore, studies suggest that YTHDF1 enhances protein synthesis in a neuronal-stimulus-dependent manner, which is essential for the formation of long-term memories [19]. These findings underscore the importance of m6A in cognitive function and open up new avenues for understanding the molecular mechanisms underlying age-related cognitive decline and the development of potential therapeutic interventions.

Neural cell adhesion molecule 1 (NCAM1), a pivotal mediator of synaptic plasticity and memory consolidation, belongs to the immunoglobulin superfamily and regulates cell–cell interactions through homophilic and heterophilic binding [20,21]. Beyond its role in structural adhesion, NCAM1 orchestrates activity-dependent synaptic remodeling by modulating signaling cascades involving Fyn kinase, ERK/MAPK, and CREB, thereby influencing long-term potentiation (LTP) and spine dynamics [22]. Age-related decline in NCAM1 correlates robustly with synaptic fragility and cognitive impairment in both humans and animal models [23,24]. Aging brains exhibit reduced NCAM1 levels in the hippocampus and prefrontal cortex, paralleling deficits in spatial memory and associative learning [25,26]. However, whether the age-related decline in NCAM1 expression is regulated by m6A modifications and whether targeting this regulatory axis could mitigate cognitive decline remain unknown.

In this study, we investigated the roles of METTL3 and IGF2BP1 in regulating *Ncam1* expression and cognitive function during aging. Using a combination of molecular, cellular, and behavioral approaches in the SAMP8 mouse model of accelerated aging, we demonstrate that both METTL3 and IGF2BP1 overexpressions enhance cognitive performance through m6A-dependent regulation of Ncam1. Our findings reveal distinct yet complementary roles for these m6A regulators in spatial learning and memory retention, providing new insights into the molecular mechanisms of age-related cognitive decline and identifying potential therapeutic targets for cognitive disorders associated with aging.

## 2. Materials and Methods

### 2.1. Animals and Cell Lines

The male SAMP8 and SAMR1 mice used in this study were obtained from Beijing Jinmuyang Animal Company (Beijing, China) [SYXK (Beijing) 2025-0011]. The animals were maintained in environmentally controlled rooms with a humidity of 60% ± 10% and a temperature of 24 °C ± 1 °C. They were also subjected to a 12-h light–dark cycle. Standard diet and water were available to the animals. Prior to the commencement of experiments, the mice were acclimated to the laboratory environment for a period of 7 days. All behavioral tests were performed during the light phase. Ethical approval for the animal experiments was obtained from the Animal Care and Use Committee of the Chinese Astronaut Research and Training Center (Reference Number: ACC-IACUC-2021-031, Approval Date: 27 December 2021). HT22 cells, which were obtained from the Shanghai Cell Bank in Shanghai, China, were cultivated in DMEM medium (Gibco, Grand Island, NY, USA) supplemented with 10% fetal bovine serum (Gibco) at 37 °C and 5% CO_2_.

### 2.2. Morris Water Maze Test

The experimental procedure comprises two distinct phases: the positioning navigation training phase and the spatial exploration phase. During the 5-day positioning navigation training period, the animals were introduced into the pool (120 cm in diameter and 40 cm in height) from various quadrants twice daily. A stationary platform was submerged 1.5 cm beneath the water surface. The spatial exploration test was conducted on the 6th day, with the platform removed from the pool. Only age-matched male mice were used for behavioral tests. Animals were randomly assigned to experimental groups prior to experiments. Behavioral performance evaluation and data analysis were conducted by investigators blinded to animal group assignments.

### 2.3. Novel Object Recognition (NOR) Test

Prior to the commencement of the familiarization and testing phases, animals were permitted to explore the experimental test box (dimensions: 45 cm length × 45 cm width × 30 cm height) once daily for a 3-day adaptation period, with each session lasting 10 min. During the familiarization phase, two identical objects were positioned symmetrically on one side of the chamber, and animals were allowed to explore for 5 min. Following a 30-min inter-session interval, one of the familiar objects was either replaced with a novel object or relocated to the contralateral position. To prevent potential biases in the experimental outcomes due to positional preferences of the animals, the familiar and novel positions were counterbalanced during the test period. The animals’ memory capacity was assessed using the discrimination index (DI). The DI is calculated using the formula: DI = (TN − TF)/(TN + TF) × 100%, where TN represents the exploration time of the novel object or the object in the new position, and TF denotes the exploration time of the familiar object.

### 2.4. Western Blotting Analysis

The hippocampal tissue was homogenized in protein lysis buffer (huaxingbio, Beijing, China) and lysed completely for 30 min. Subsequently, the homogenate was centrifuged at 13,000 *g* for 15 min at 4 °C, and the supernatant was collected. The protein concentration was determined using BCA protein assay kits (Thermo Fisher Scientific, Waltham, MA, USA). The protein samples were then mixed with a 5× loading buffer and heated at 100 °C for 5 min. Proteins were separated by SDS-PAGE and transferred onto a PVDF membrane (Merck Millipore, Darmstadt, Germany). The membrane was blocked with 5% non-fat milk in Tris Buffered Saline Tween (TBST) (Solarbio, Beijing, China) for 1 h at room temperature. The membranes were incubated with primary antibodies, including CREB/p-CREB (1:1000, Cell Signaling Technology, CST, Danvers, MA, USA), METTL3 (1:1000, CST), IGF2BP1 (1:1000, CST), IGF2BP2 (CST), NCAM1 (1:2000, CST), α-TUBULIN (1:1000, Proteintech, Chicago, IL, USA), and GAPDH (1:1000, Proteintech), overnight at 4 °C. This was followed by incubation with HRP-conjugated secondary antibodies for 1 h at room temperature. The protein bands were visualized using an ECL kit (Merck Millipore). The intensity of the bands was analyzed using ImageJ software (version 1.8.0.345, National Institutes of Health, Bethesda, MD, USA) to quantify the band density.

### 2.5. Nissl’s Staining

In each group, three paraffin sections were subjected to dewaxing with xylene and then rehydrated through a series of graded ethanol solutions (70%, 95%, and 100%), followed by final rehydration in distilled water. The staining procedure was carried out using a Nissl staining kit (Jiancheng Biology, Nanjing, China). High-resolution images of the stained sections were captured using a microscope slide scanner (Pannoramic 250, 3D Histech Ltd., Budapest, Hungary). The integrated optical density (IOD) of Nissl bodies in each group was subsequently quantified and analyzed using NIH ImageJ software.

### 2.6. RT-qPCR

Reverse transcription of RNA was performed using the PrimeScript RT reagent Kit with gDNA Eraser (TaKaRa, Dalian, China). For RT-qPCR, the TB Green Premix Ex Taq II (TaKaRa) was utilized. The mRNA expression levels were normalized to GAPDH. The sequences of the oligonucleotides used are provided in the Supplementary Table S1.

### 2.7. MeRIP-qPCR

Total RNA was extracted using the Trizol reagent (Invitrogen, Carlsbad, CA, USA). A portion of the total RNA (50 ng) was reserved as input material. The remaining RNA (3 μg) was subjected to m6A-immunoprecipitation using an anti-N6-methyladenosine (m6A) antibody (Synaptic Systems, Göttingen, Germany) in a 500 μL reaction volume containing IP buffer (150 mM NaCl, 0.1% NP-40, 10 mM Tris, pH 7.4, and 100 U RNase inhibitor). The m6A-containing RNAs were captured using Protein G beads (MCE, Monmouth Junction, NJ, USA) and subsequently eluted three times with elution buffer (5 mM Tris-HCl, pH 7.5, 1 mM EDTA, pH 8.0, 0.05% SDS, and 20 mg/mL Proteinase K). Both 2 ng of the total RNA and 2 ng of the m6A-immunoprecipitated RNA were used as templates for RT-qPCR, following the procedures described earlier. The m6A level was normalized to the input.

### 2.8. RNA Epitranscriptomic Microarray

Total RNAs were subjected to immunoprecipitation using an m6A antibody. This process yielded two distinct fractions: the immunoprecipitated “IP” fraction, which is enriched in m6A-methylated RNAs, and the supernatant “Sup” fraction, which contains unmodified RNAs. Both the “IP” and “Sup” RNAs were subsequently amplified into cRNAs and labeled with Cy5 and Cy3, respectively, using the Arraystar Super RNA Labeling Kit (Arraystar, Rockville, MD, USA). The Cy3-and Cy5-labeled cRNAs were combined and hybridized to Arraystar Mouse mRNA and lncRNA Epitranscriptomic Arrays (8 × 60 K, Arraystar) at 65 °C for 17 h in an Agilent Hybridization Oven (Agilent Technologies, Santa Clara, CA, USA). Following hybridization, the slides were washed and scanned using the Agilent Scanner G2505C (Agilent Technologies). Data extraction was performed using Agilent Feature Extraction software version 12.2 (Agilent Technologies). Probes with “P” or “M” QC flags in all three samples were retained for further analysis. The IP (Cy5-labeled) intensities were normalized relative to RNA spike-in calibration controls to represent the “m6A quantity” of the transcript, allowing for comparison across samples. Differentially m6A-methylated mRNAs were identified based on “m6A quantity” that met the fold change and statistical significance cutoffs and were compiled for further study.

Raw signal data were extracted using Agilent Feature Extraction software. For quality-control filtering, only probes assigned “P” (present) or “M” (marginal) QC flags across all three biological replicates were retained for downstream analysis. IP-derived Cy5 signal intensities were normalized against RNA spike-in calibration controls to calculate the relative m6A quantity for each transcript, enabling cross-sample comparison of m6A modification levels.

Differentially m6A-methylated mRNAs were screened according to predefined fold-change and statistical significance thresholds. Briefly, transcripts with |fold change| ≥ 1.5 and *p*-value < 0.05 were defined as significantly differentially m6A-modified. To prioritize functional candidate genes, we intersected transcripts exhibiting significant m6A alterations with genes showing concomitant differential mRNA expression from the parallel expression array dataset. Among these overlapping candidates, Ncam1 stood out because it displayed both decreased m6A methylation and reduced transcript abundance in SAMP8 mice relative to SAMR1 controls, and it has well-documented functions in hippocampal synaptic plasticity and cognitive regulation. This multi-step prioritization workflow led to the selection of Ncam1 as the core target for subsequent mechanistic validation.

### 2.9. The Gene Ontology Analysis

For Gene Ontology (GO) analysis, the Gene Ontology database (http://www.geneontology.org)) is utilized. This database encompasses three main categories: Biological Process (BP), Cellular Component (CC), and Molecular Function (MF). The significance of GO term enrichment is typically assessed using Fisher’s exact test, with *p*-values converted into enrichment scores (−log10(P)). A lower *p*-value or a higher enrichment score suggests more significant enrichment. In general, a *p*-value below 0.05 is regarded as statistically significant.

### 2.10. Vector Construction and Cell Transfection

The plasmids designed to overexpress IGF2BP1 and Ncam1 were developed by Fenghui Biotech (Changsha, China). In the case of the Ncam1 mutant version, adenine (A) to cytosine (C) mutations were introduced using the KOD Plus enzyme (Vazyme, Nanjing, China). The transfection of siRNAs and plasmids was conducted using the RNAiMAX and Lipofectamine 3000 kits (Invitrogen), following the manufacturer’s instructions. The sequences of the siRNAs utilized are provided in the Supplementary Table S1.

### 2.11. D-galactose Treatment Assay

D-galactose (D-gal) (Sigma-Aldrich, St. Louis, MO, USA) was dissolved in DMEM. The solution was thoroughly mixed in a water bath maintained at 37 °C to ensure complete dissolution. HT22 cells were treated with gradient D-gal concentrations (50 mM, 100 mM, and 200 mM), with the 0 mM group serving as the vehicle control. Among these concentrations, 200 mM D-gal was applied for the formal rescue experiments. After 48 h of treatment, the cells were harvested for subsequent experiments.

### 2.12. mRNA Stability Assay

Transcription inhibition was performed by adding actinomycin D (Sigma-Aldrich) (5 μg/mL) to block de novo RNA synthesis. Cells were harvested at 0 h, 6 h, and 12 h after actinomycin D treatment. Total RNA was extracted at each time point, and the remaining Ncam1 mRNA levels were quantified by qRT-PCR. GAPDH was used as an internal reference. Relative mRNA residual levels were normalized to the 0 h time point. The mRNA half-life was calculated based on the exponential decay curve to evaluate the effect of experimental interventions on Ncam1 mRNA stability.

### 2.13. Immunofluorescence Staining

Cells were fixed with 4% paraformaldehyde (Sigma-Aldrich) for 20 min at room temperature, permeabilized with 0.3% Triton X-100 (Sigma-Aldrich) for 15 min, and blocked with 5% bovine serum albumin (BSA) (Sigma-Aldrich) for 1 h to eliminate non-specific binding. Subsequently, cells were incubated with primary antibodies of NCAM1 (1:50), IGF2BP1 (1:50), or METTL3 (1:50) overnight at 4 °C. After thorough washing with PBS (Solarbio, Beijing, China), cells were incubated with corresponding fluorophore-conjugated secondary antibodies for 1 h at room temperature in the dark. Nuclei were counterstained with DAPI (Sigma-Aldrich). Fluorescence images were captured using a laser confocal scanning microscope (Carl Zeiss, Jena, Germany) under consistent exposure parameters across all groups.

### 2.14. Cell Viability Assay (CCK-8)

Cell viability was evaluated using the Cell Counting Kit-8 (CCK-8) (Solarbio) assay. HT22 cells were seeded into 96-well plates at an appropriate density and overexpression of NCAM1, IGF2BP1 or METTL3 through transduction for 12 h and then subjected to D-galactose (200 mM) treatment for 48 h. After treatment, 10 μL of CCK-8 reagent was added to each well containing 100 μL of culture medium. The plates were incubated at 37 °C for 1–2 h away from light. The absorbance value at 450 nm was measured using a microplate reader (BioTek Instruments, Winooski, VT, USA). Blank wells containing medium without cells were set for background correction. Relative cell viability was calculated by normalizing the absorbance of experimental groups to the control group.

### 2.15. Image Quantification Analysis

All immunofluorescence and Western blot band intensity analyses were performed using ImageJ software with unified analytical parameters. For immunofluorescence images, the mean fluorescence intensity of positively stained regions was quantified in at least five random fields per sample. For Western blots, the gray value of each target band was normalized to the corresponding internal reference band to obtain relative protein expression levels. All quantitative analyses were conducted with identical threshold settings, image brightness, and contrast parameters across different experimental groups to ensure quantification consistency and objectivity.

### 2.16. Immunohistochemistry (IHC)

The whole brain was collected and fixed in a 4% paraformaldehyde (Sigma-Aldrich) solution for a specific duration, such as 24 h. Following fixation, the tissue was transferred into 70% ethanol for dehydration. Subsequently, the tissue was embedded and sectioned. The slides were placed in xylene for deparaffinization, repeating the process 2–3 times, with each cycle lasting approximately 5 min. The slides were then rehydrated by passing them through a series of graded ethanol solutions (e.g., 100%, 95%, and 70% ethanol) for a few minutes each. A blocking solution (1% BSA (Sigma-Aldrich) in PBS) was applied to the slides, and they were incubated for about 1 h at room temperature to prevent non-specific binding of antibodies. The diluted primary antibody solution was applied to the tissue sections and incubated overnight at 4 °C. The diluted secondary antibody solution was then applied to the tissue sections and incubated for 1–2 h at room temperature. Finally, DAB substrate kit (Vector Laboratories, Burlingame, CA, USA) was used for visualization.

### 2.17. RNA Immunoprecipitation

RNA Immunoprecipitation (RIP) experiments were conducted using the Magna RIP RNA-Binding Protein Immunoprecipitation Kit (Merck Millipore) following the manufacturer’s instructions. The co-precipitated RNA was then detected by RT-qPCR to analyze the binding of RNA-binding proteins to specific RNA molecules.

### 2.18. Luciferase Assay

For the luciferase assay, plasmids containing the target promoter region and downstream reporter genes (firefly luciferase and Renilla luciferase) were designed and constructed. HT22 cells were plated in a 48-well plate and allowed to adhere overnight. The cells were then transfected with the constructed plasmid. After 24 h, the cells were washed with PBS. The cells were lysed for 20 min using a lysis buffer, and the lysate was transferred to a white opaque 96-well plate. The luciferase reagent and Stop&Glo reagent solution (Promega, Madison, WI, USA) were prepared and used for detection. The data were analyzed by calculating the ratio of the firefly luciferase activity to the Renilla luciferase activity.

### 2.19. AAV Vectors and Hippocampal Stereotaxic Injection

Four recombinant adeno-associated virus (AAV) vectors were utilized in this study, namely the empty control vector (AAV-vector), AAV-Mettl3, AAV-Igf2bp1, and AAV-Ncam1. All overexpression constructs were generated using the pHBAAV-CMV-MCS-3flag-T2A-ZsGreen backbone. Transgene expression in these vectors is driven by the cytomegalovirus (CMV) promoter, with ZsGreen acting as a fluorescent reporter. ZsGreen fluorescence signals were used to assess AAV transduction efficiency in the mouse hippocampus. Plasmid maps for all three overexpression constructs, as well as their full transgene sequences, are provided in the Supplementary Materials.

The stereotaxic injection technique was employed to target the hippocampal region with AAV vectors. Mice were anesthetized using isoflurane (Sigma-Aldrich) and then fixed in a stereotaxic apparatus (Stoelting Co., Wood Dale, IL, USA). A small incision was created on the scalp to reveal the skull, and the coordinates for the target region were set to the CA1 area (Bregma: anteroposterior-2.3 mm, lateral ±1.8 mm, ventral 2.0 mm). The AAV solutions, including AAV-vector, AAV-Mettl3, AAV-Igf2bp1, and AAV-Ncam1, were loaded into a microinjector (World Precision Instruments, Sarasota, FL, USA) fitted with a fine glass pipette. The AAV solution was carefully infused into the target area using the microinjector, and the pipette was maintained in position for a brief period to facilitate diffusion and reduce backflow. After the injection, the pipette was carefully withdrawn, and the incision was closed with sutures (Ethicon, Somerville, NJ, USA).

### 2.20. Transmission Electron Microscopy

The SAMP8 and SAMR1 mice were subjected to decapitation, and their brains were promptly removed. The hippocampal tissue was carefully dissected on ice and sectioned into small pieces of approximately 1 mm^3^. These tissue pieces were immediately transferred into a 2.5% glutaraldehyde fixative solution (Sigma-Aldrich) and fixed at 4 °C overnight. The samples were then rinsed three times with phosphate buffer (0.1 M, pH 7.0), followed by fixation in a 1% osmium acid solution (Sigma-Aldrich) for 2 h. Afterward, the samples were rinsed again with phosphate buffer three times and dehydrated using a graded ethanol series. Finally, the samples were embedded in epoxy resin (Electron Microscopy Sciences, Hatfield, PA, USA), polymerized at 80 °C for 24 h, and cut into 100 nm ultra-thin sections. These sections were stained with uranyl acetate and lead citrate (Sigma-Aldrich) and observed under a transmission electron microscope (Hitachi, Tokyo, Japan).

### 2.21. Statistical Analysis

Statistical analyses were performed using GraphPad Prism software (version 10.2.0, GraphPad Software, San Diego, CA, USA). For comparisons between two groups, the unpaired Student’s *t*-test was applied as appropriate. For multiple-group comparisons, one-way analysis of variance (ANOVA) followed by Tukey’s post hoc test was used. For Morris water maze training-latency data collected across successive training days, repeated-measures two-way ANOVA was performed to evaluate the effects of group and training day, with Bonferroni post hoc correction for multiple comparisons. Other multiple-comparison analyses were corrected using the false discovery rate (FDR). Results are presented as mean ± standard error of the mean (SEM) or mean ± standard deviation (SD), or fold change (FC) relative to the corresponding control group. The significance of Gene Ontology (GO) enrichment terms was assessed using Fisher’s exact test with FDR adjustment for multiple comparisons. A *p*-value or FDR < 0.05 was defined as statistically significant.

## 3. Results

### 3.1. Spatial Memory Deficits in SAMP8 Mice

To assess the spatial learning and memory deficits associated with aging, we performed the Morris Water Maze (MWM) test on two groups of 6-month-old SAMR1 and senescence-accelerated SAMP8 mice. During the initial 5-day training period, we detected a substantial main effect of day on the latency to reach the platform, which demonstrated the progressive learning that occurred throughout the training duration. Consistent with our hypothesis, SAMP8 mice exhibited longer latencies to find the platform when compared to SAMR1 mice (Figure S1A). On day 6, a probe trial was conducted with the hidden platform removed, allowing mice to swim freely for 60 s to assess their spatial reference memory. Although no significant differences in platform swimming speed were observed between the two groups during this period (Figure S1B), the number of platform crossings (Figure S1C), swimming distance (Figure S1D), and time spent in the target quadrant (Figure S1E) were significantly reduced in SAMP8 mice. Additionally, the percentage of swimming distance and time in the target quadrant was also lower in the SAMP8 group (Figure S1F,G). Collectively, these results demonstrate that SAMP8 mice exhibit impaired spatial learning and memory compared to SAMR1 mice.

### 3.2. m6A Modifications and Transcriptome Dynamics in SAMP8 Mice

**Figure 1:**
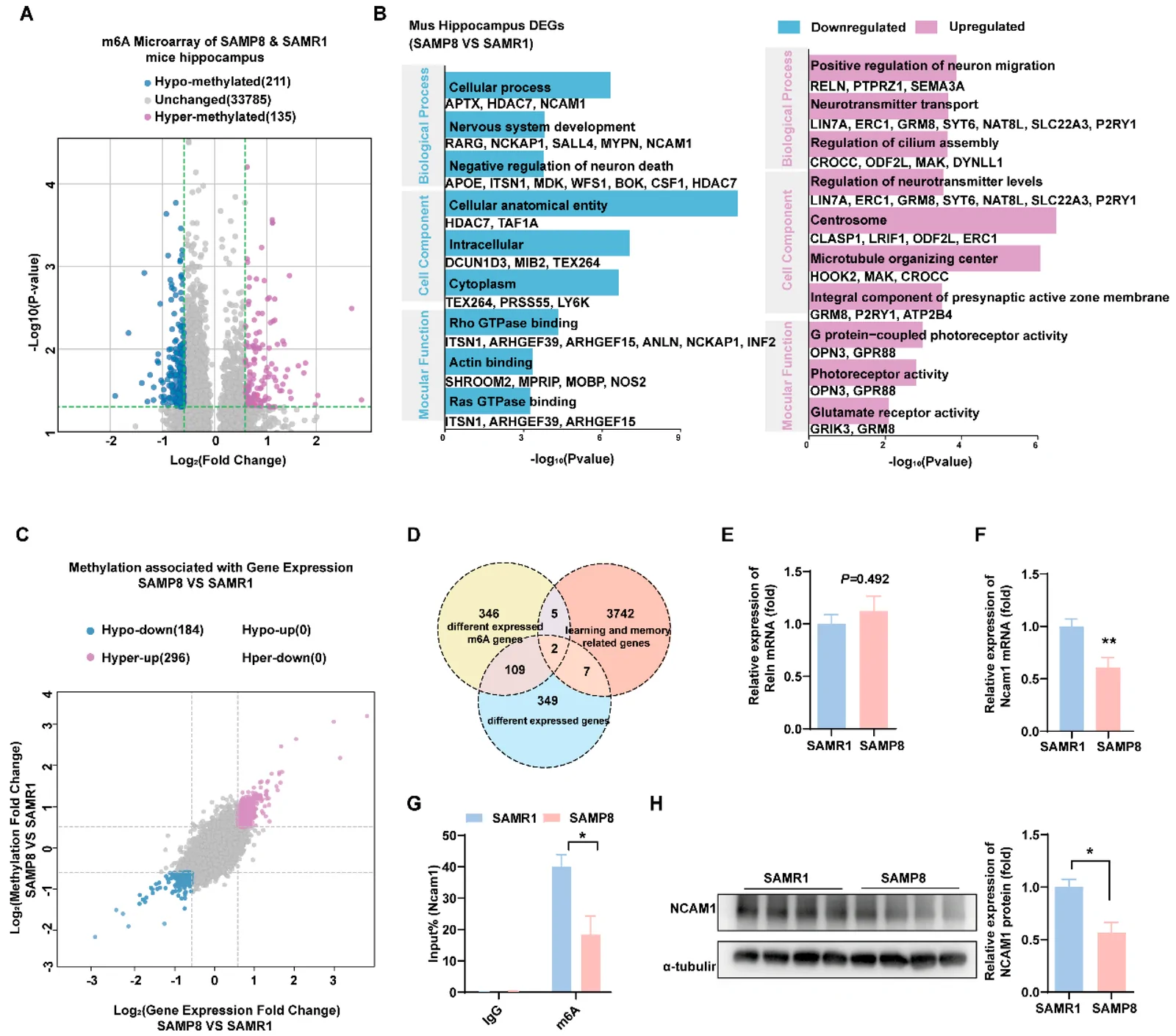
**The integrated analysis of transcriptomic and epitranscriptomic data reveals a downregulation of Ncam1 within the hippocampus of SAMP8 mice:** (**A**) Volcano plots of the different expressed m6A-modified genes between SAMP8 and SAMR1 mice hippocampus. (**B**) Gene Ontology (GO) enrichment analysis of differentially expressed m6A-modified genes. (**C**) Differently expressed m6A-modified genes and differently expressed genes between SAMP8 and SAMR1 mice were jointly analyzed, and a four-dimensional quadrant map was generated. Hypo-down: hypomethylated genes with downregulated. Hypo-up: hypomethylated genes with upregulated expression; Hyper-up: hypermethylated genes with upregulated expression; Hyper-down: hypermethylated genes with downregulated expression. (**D**) Venn diagram of differentially expressed genes and differentially expressed m6A genes combined with the learning and memory-related genes. (**E**) Expression of *Reln* mRNA levels in the hippocampal tissues of SAMR1 and SAMP8 mice (n = 6 per group). (**F**) Expression of *Ncam1* mRNA levels in the hippocampal tissues (n = 6). (**G**) m6A level of *Ncam1* in the hippocampus of SAMR1 and SAMP8 mice (n = 6). (**H**) Western blot to assess NCAM1 protein level in SAMR1 and SAMP8 mice hippocampus (n = 4); α-tubulin was the internal control. (* *p* < 0.05, ** *p* < 0.01).

To characterize the transcriptome-wide dynamics of m6A modifications in SAMP8 mice, we performed m6A epitranscriptomic microarray analysis on hippocampal tissues from both 6-month-old SAMR1 and SAMP8 mice. Differentially expressed m6A-modified genes were identified using a threshold of FDR < 0.05 and |log2 fold change| ≥ 0.58 (equivalent to |fold change| ≥ 1.5). Comparative analysis revealed 346 m6A-modified transcripts with differential expression in SAMP8 compared to SAMR1 mice, including 135 hypermethylated and 211 hypomethylated genes. A volcano plot illustrating the distribution of these m6A-modified genes is shown in Figure 1A. GO enrichment analysis of the differentially m6A-modified mRNAs highlighted significant dysregulation of nervous-system-relevant processes—most notably, nervous system development and neurotransmitter transport—in SAMP8 mice versus SAMR1 controls (Figure 1B). These findings implicate m6A epitranscriptomic dysregulation in age-related neuronal dysfunction. Utilizing transcriptome data analysis, we identified a total of 349 differentially expressed mRNAs under stringent statistical thresholds (false discovery rate [FDR] < 0.05, absolute log2 fold change [|log2FC|] ≥ 0.58). These differentially expressed mRNAs included 185 upregulated and 164 downregulated transcripts, as depicted in the volcano plot (Supplementary Figure S2A). An integrative analysis of the crosstalk between the transcriptome and epitranscriptome (Figure 1C) unveiled an age-dependent coordination. Specifically, transcripts with m6A hypomodification displayed a corresponding downregulation of mRNA levels. In contrast, those with m6A hypermodification exhibited concurrent upregulation. This pattern indicates a genome-wide positive correlation between m6A stoichiometry and transcript abundance.

To identify central mediators of m6A-driven cognitive decline, we implemented a multi-tiered screening strategy: transcriptomic, epitranscriptomic criteria and learning/memory-associated genes (GO:0007611) (Figure 1D). This integrated approach prioritized two high-confidence candidates: RELN (Reelin, NM_011261) and NCAM1 (neural cell adhesion molecule 1, ENSMUST00000114476), both of which exhibited m6A hypomodification and downregulated mRNA expression in the microarray dataset. Then we examined the expression of RELN and NCAM1 in the hippocampal tissues of SAMP8 and SAMR1. As shown in Figure 1E, RELN expression showed no significant alteration in SAMP8 mice compared to SAMR1 controls (*p* = 0.49). In contrast, NCAM1, a multifunctional regulator of neurodevelopmental processes, including axonal pathfinding, synaptic plasticity maintenance, and spatial memory consolidation, demonstrated coordinated m6A modification changes coupled with transcriptional downregulation. And experimental validation revealed decreases in both *Ncam1* mRNA (RT-qPCR: 0.61-fold, *p* = 0.008, n = 6; Figure 1F) and m6A enrichment (MeRIP-qPCR: 0.45-fold, *p* = 0.01; Figure 1G) in SAMP8 hippocampus. This discrepancy was corroborated at the protein level, with Western blotting showing a 44% reduction in NCAM1 expression (Figure 1H). These results indicate that downregulation of NCAM1 is an important mediator of cognitive decline in SAMP8 mice.

### 3.3. METTL3-Mediated NCAM1 Transcriptional Attenuation in Aging Neuronal Cells

**Figure 2:**
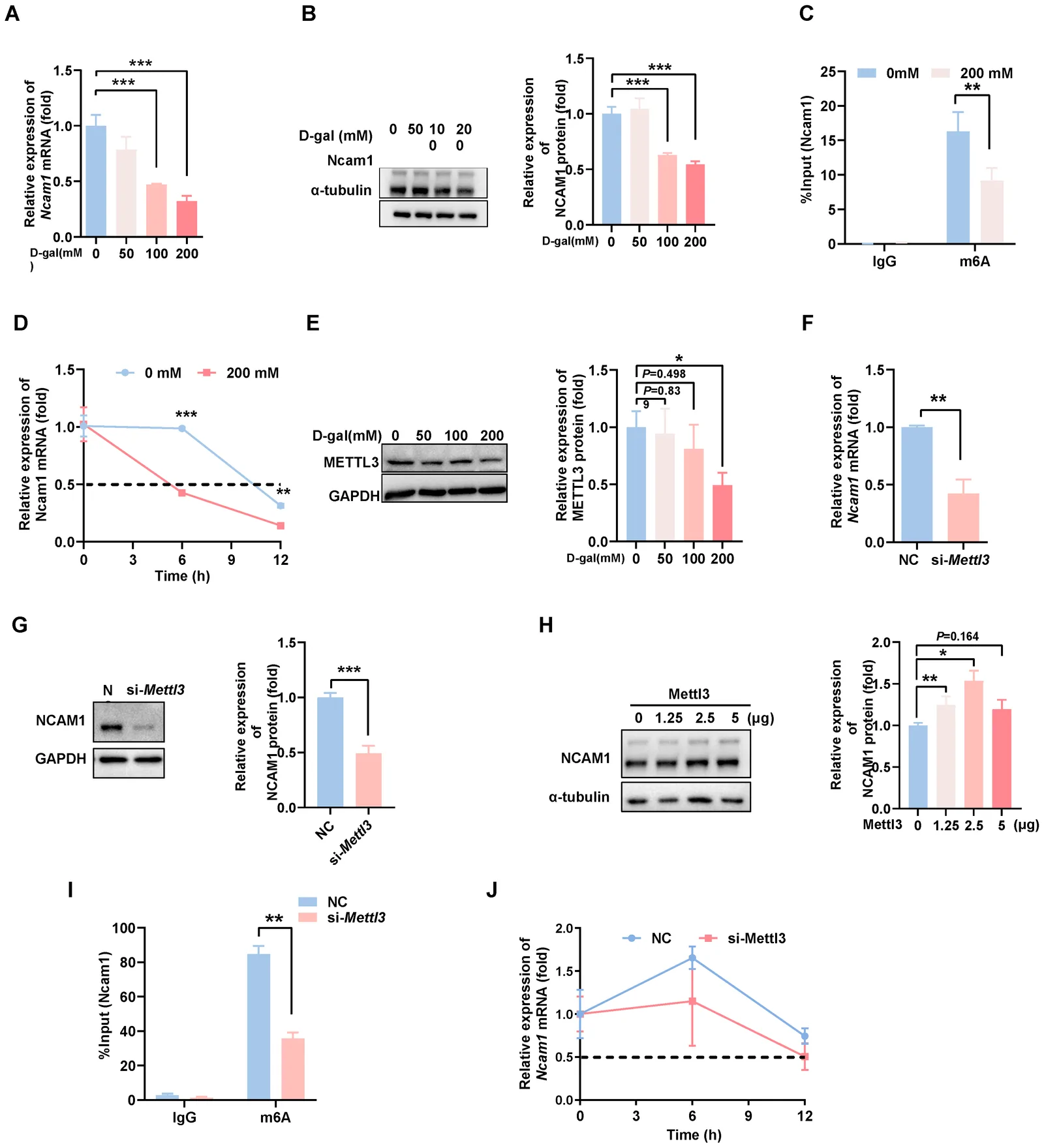
**Mettl3 orchestrates declining Ncam1 and its m6A modification in senescent cells:** (**A**,**B**) HT22 cells were treated with D-gal at 50, 100, and 200 mM respectively for 48 h, and Ncam1 mRNA (**A**,**B**) expression were examined (n = 3). (**C**) MeRIP-qPCR to assess the m6A modification level of *Ncam1* in senescent HT22 cells (n = 3). (**D**) Stability of *Ncam1* mRNA in senescent HT22 cells (n = 3). (**E**) Western blot to assess the protein expression levels of METTL3 and the quantitative analysis (n = 3). (**F**) mRNA expression of *Ncam1* after *Mettl3* knockdown (n = 6). (**G**) Western blot and quantitative analysis of the protein expression of NCAM1 after *Mettl3* knockdown (n = 3). (**H**) Western blot and quantitative analysis of the protein expression of NCAM1 after *Mettl3* overexpression (n = 3). (**I**) MeRIP-qPCR detected the m6A level of *Ncam1* after *Mettl3* knockdown (n = 3). (**J**) mRNA stability of *Ncam1* after *Mettl3* knockdown (n = 3). (* *p* < 0.05, ** *p* < 0.01, *** *p* < 0.001).

To further elucidate the role of m6A-modified Ncam1 in aging-related cognitive decline, we established a D-galactose (D-gal)-induced neuronal aging model using HT22 mouse hippocampal neuronal cells. Ncam1 expression at both the mRNA and protein levels was significantly reduced in D-gal-treated HT22 cells (Figure 2A,B). Methylated RNA immunoprecipitation quantitative PCR (MeRIP-qPCR) analysis showed that the level of m6A modification on *Ncam1* mRNA was significantly decreased during cellular senescence (Figure 2C). These *in vitro* results are consistent with our previous observations in SAMP8 mice (Figure 1H).

To determine whether reduced m6A modifications mediate Ncam1 downregulation during cellular senescence, we assessed the stability of *Ncam1* mRNA. The results revealed that m6A hypomodification significantly decreased the half-life of *Ncam1* transcripts in D-galactose-treated cells (Figure 2D), thereby highlighting the role of m6A modifications in stabilizing *Ncam1* mRNA. Given that METTL3, an m6A methyltransferase, is implicated in the aging process and plays a pivotal role in regulating learning and memory [27], we explored the effects of D-gal treatment on METTL3 expression. Intriguingly, our immunoblotting analysis showed a dose-dependent decrease in METTL3 protein expression following treatment with varying concentrations of D-gal (Figure 2E). To investigate the potential regulatory relationship between METTL3 and Ncam1 expression, we systematically examined their molecular interplay through gain- and loss-of-function experiments. Initial siRNA-mediated METTL3 knockdown (si-*Mettl3*) in HT22 cells resulted in a marked decrease in both Ncam1 mRNA (*p* < 0.01) and protein levels (*p* < 0.001) compared to negative controls (NC), as quantified through RT-qPCR and immunoblotting analyses (Figure 2F,G and Figure S3A,B). Conversely, vector-mediated METTL3 overexpression demonstrated a concomitant upregulation of NCAM1 protein expression (Figure 2H and Figure S3C). Subsequently, we performed MeRIP-qPCR to mechanistically link these observations with METTL3’s catalytic function. The analysis revealed a significant 42% reduction in m6A modification levels and decreased stability of *Ncam1* mRNA following METTL3 depletion (Figure 2I,J), establishing a direct association between METTL3-mediated m6A deposition and Ncam1 expression regulation. In summary, these findings demonstrate that D-gal-induced downregulation of METTL3 impairs Ncam1 expression through reduced m6A modification in HT22 cells, revealing a crucial METTL3-m6A-Ncam1 regulatory axis underlying aging-related cellular phenotypes.

### 3.4. IGF2BP1 Regulate NCAM1 Expression via m6A Modification

**Figure 3:**
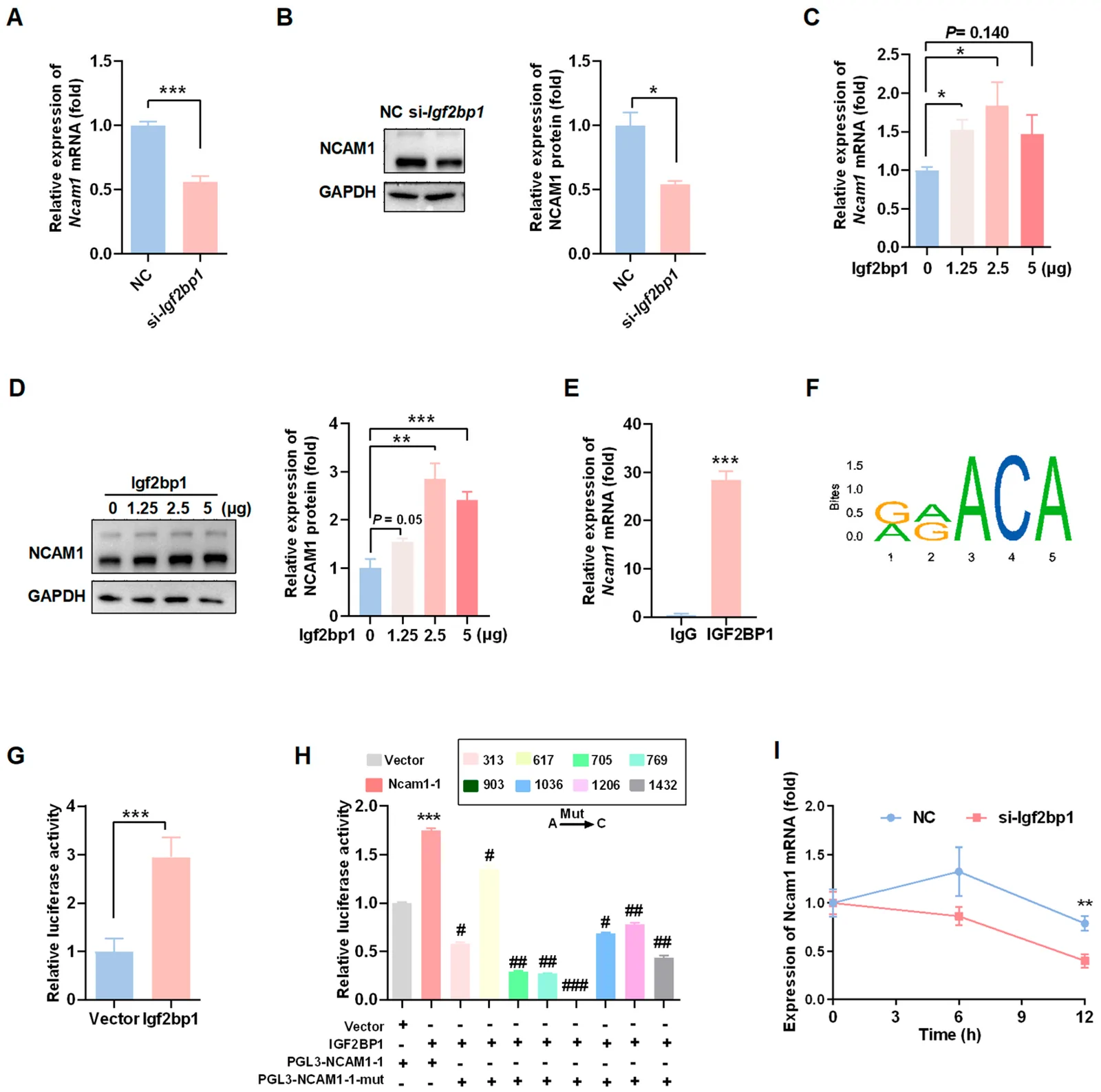
**The expression of Ncam1 was orchestrated by the m6A reader IGF2BP1:** (**A**) mRNA expression of *Ncam1* after *Igf2bp1* knockdown (n = 6). (**B**) Western blot and quantitative analysis of the protein level of NCAM1 after *Igf2bp1* knockdown (n = 3). (**C**) mRNA expression of *Ncam1* after *Igf2bp1* over expressed (n = 6). (**D**) Western blot to assess the protein expression levels of NCAM1 after *Igf2bp1* overexpression and the quantitative analysis of NCAM1 protein expression (n = 3). (**E**) RIP-qPCR detected *Ncam1* as a binding molecule downstream of IGF2BP1 (n = 3). (**F**) The sequencing analysis identified the m6A modification sites of Ncam1. (**G**) Luciferase analysis to assess the regulatory effects of Igf2bp1 on Ncam1 (n = 6). (**H**) Luciferase assay to evaluate the binding affinity of IGF2BP1 to *Ncam1* mRNA following targeted mutation at each m6A modification site (n = 6). (**I**) Stability of *Ncam1* mRNA following treatment with si-*Igf2bp1* in HT22 cells (n = 3) (* *p* < 0.05, ** *p* < 0.01, *** *p* < 0.001 compared with the respective control group, # *p* < 0.05, ## *p* < 0.01, ### *p* < 0.001 compared with the PGL3-NCAM1-1 group).

Given the critical role of m6A readers in mediating mRNA fate decisions, we explored whether specific readers might recognize and stabilize m6A-modified *Ncam1* transcripts. Previous studies reported that IGF2BPs can recognize thousands of m6A-modified transcripts and enhance the stability and translation of these mRNAs, with *Ncam1* mRNA identified as a potential target of both IGF2BP1 and IGF2BP3 through high-throughput sequencing analyses [28]. Therefore, we hypothesized that IGF2BP1/3 might act as molecular bridges to stabilize m6A-modified Ncam1 transcripts. To validate the regulatory role of IGF2BP1 in *Ncam1* expression, we performed loss-of-function experiments by silencing *Igf2bp1* using small interfering RNA (si-*Igf2bp1*). IGF2BP1 knockdown significantly reduced both Ncam1 mRNA and protein levels compared to scrambled siRNA controls (Figure S3D,E and Figure 3A,B). Conversely, gain-of-function studies via an IGF2BP1 overexpression plasmid (pcDNA3.1-IGF2BP1) resulted in marked upregulation of Ncam1 at both transcriptomic and proteomic levels relative to the empty vector control (Figure S3F and Figure 3C,D). Notably, siRNA-mediated knockdown of *Igf2bp3* did not significantly alter *Ncam1* mRNA levels compared to negative controls (Figure S3G,H), indicating that IGF2BP3 does not regulate Ncam1 transcription.

RNA immunoprecipitation (RIP)-qPCR analysis using an anti-IGF2BP1 antibody confirmed a specific interaction between IGF2BP1 and *Ncam1* mRNA, with significantly higher enrichment observed in IGF2BP1 immunoprecipitates compared to IgG controls (Figure 3E). To identify the specific m6A modification sites on *Ncam1* mRNA, we analyzed the sequence of Ncam1 and mapped the m6A peak to the 3’UTR region of *Ncam1* (Figure 3F). Using this information, we constructed a luciferase reporter plasmid harboring the wild-type *Ncam1* m6A motif and performed dual-luciferase reporter assays. Functional validation revealed that IGF2BP1 preferentially binds to m6A sites, leading to significant activation of luciferase activity (Figure 3G). Furthermore, the Ncam1 transcript harbors 8 conserved m6A motifs (RRACA). To further explore the interaction between IGF2BP1 and these m6A motifs, we performed site-directed mutagenesis on these loci and subsequently measured luciferase activity. The results demonstrated that IGF2BP1 can bind to all of these m6A motifs, albeit with varying binding intensities (Figure 3H). Moreover, following the knockdown of Igf2bp1, the mRNA level of Ncam1 exhibited a time-dependent decline, indicating the decreased stability of Ncam1 mRNA (Figure 3I). In summary, our findings demonstrate that IGF2BP1 specifically recognizes and stabilizes m6A-modified Ncam1 transcripts through direct binding to conserved m6A motifs in the 3’UTR, thereby playing a critical role in regulating Ncam1 expression at the post-transcriptional level.

### 3.5. m6A-Dependent NCAM1 Regulation by METTL3/IGF2BP1 Controls CREB Activation in Neuronal Cells

**Figure 4:**
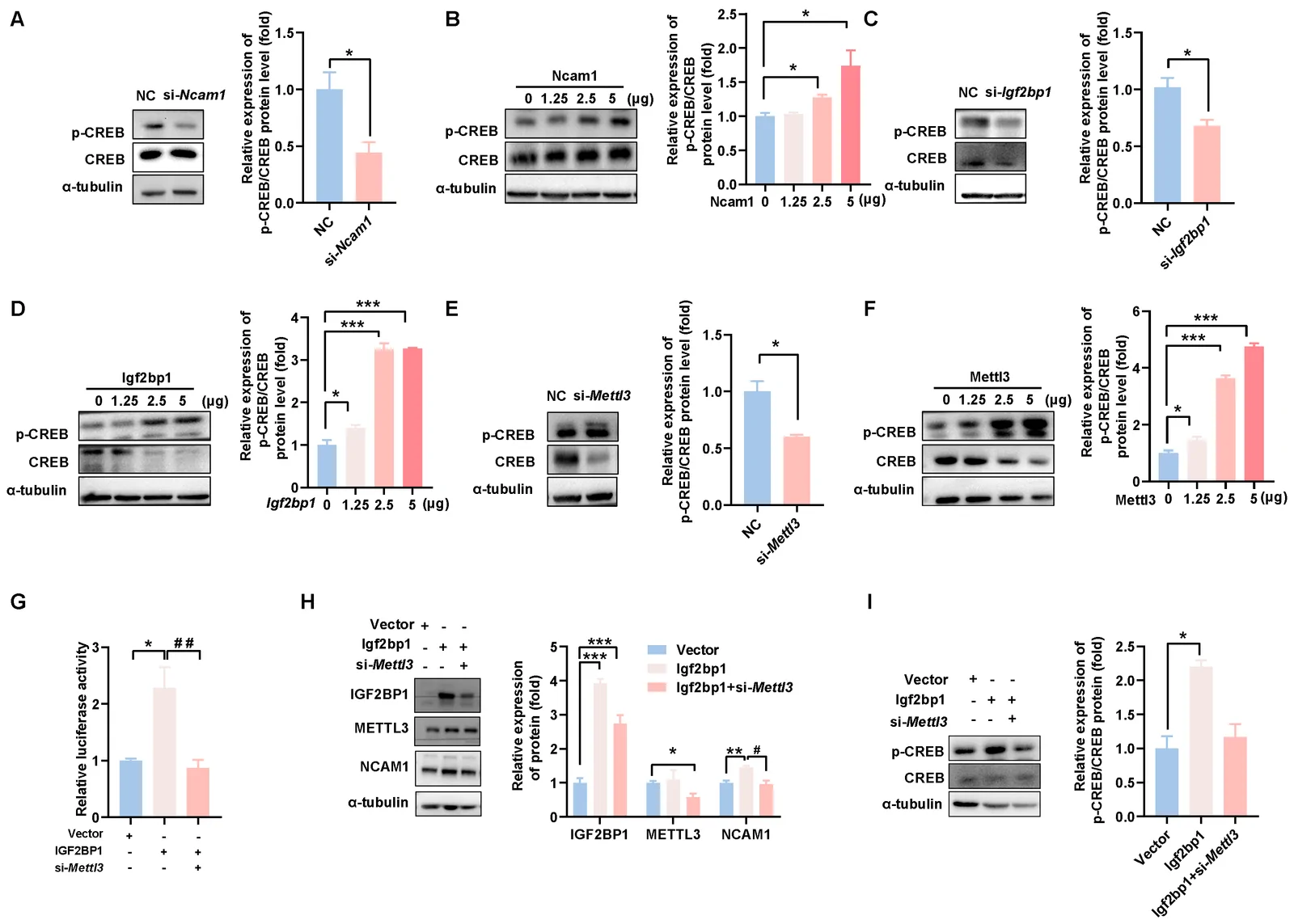
**IGF2BP1/METTL3-NCAM1 axis modulates CREB phosphorylation:** (**A**–**F**) Western blot analysis of CREB protein expression and phosphorylation levels following knockdown or overexpression of *Ncam1* (**A**,**B**), *Igf2bp1* (**C**,**D**), and *Mettl3* (**E**,**F**) (n = 3 for each condition). (**G**) Luciferase to assess the binding ability of *Ncam1* and *Igf2bp1* after co-transfection of *Igf2bp1* over expressed plasmid and siRNA of *Mettl3* (n = 6). Western blot analysis of NCAM1 (**H**) and p-CREB (**I**) protein levels following co-transfection with an Igf2bp1 overexpression plasmid and Mettl3-targeting siRNA (n = 3). (* *p* < 0.05, *** *p* < 0.001, # *p* < 0.05, ## *p* < 0.01). Note: p-CREB/CREB shows a double-band pattern; densitometric quantification was performed only on the main dominant band. The main dominant band of IGF2BP1 and NCAM1were used for densitometric quantification.

Given the established role of CREB in memory formation, emerging evidence suggests that NCAM1 deficiency disrupts CREB-mediated signaling in memory-associated brain regions [29,30]. NCAM140 isoform promotes ERK1/2 activation via the focal adhesion kinase p125fak-p59fyn-Ras signaling axis, thereby enhancing CREB phosphorylation at Ser133 [31]. To establish the functional relationship between NCAM1 and CREB signaling, we performed systematic perturbation experiments. SiRNA-mediated NCAM1 knockdown (si-*Ncam1*) in HT22 cells resulted in a 56% reduction in phosphorylated CREB (p-CREB) levels compared to negative control (NC) (*p* < 0.05, Figure 4A and Figure S3I,J), as quantified by immunoblotting and densitometric analysis. Conversely, overexpression of NCAM1 (OE-NCAM1) resulted in a dose-dependent increase in CREB phosphorylation, with the maximum induction reaching 1.7-fold at 48 h post-transduction (*p* < 0.05, Figure 4B and Figure S3K). The results of these reciprocal experiments establish a robust positive correlation between NCAM1 expression levels and CREB activation status, suggesting that NCAM1 functions upstream of CREB phosphorylation in the signaling cascade.

Consistent with these observations, RNAi-mediated depletion of either *Igf2bp1* or *Mettl3* in neuronal cells resulted in a marked reduction (60–68%) in p-CREB levels (*p* < 0.05, Figure 4C,E), as quantified by immunoblotting and normalized to total CREB. Conversely, transient transfection of IGF2BP1 or METTL3 expression plasmids elicited a dose-dependent enhancement of CREB phosphorylation, with maximal induction reaching 3.25- to 4.75-fold at 48 h post-transfection (*p* < 0.005, Figure 4D,F). These reciprocal perturbations establish IGF2BP1 and METTL3 as positive regulators of CREB signaling, potentially through their coordinated actions in m6A-mediated RNA metabolism.

We employed a dual-luciferase reporter system to mechanistically dissect the interplay between IGF2BP1 and METTL3 in regulating NCAM1 expression. Overexpression of IGF2BP1 significantly enhanced its binding affinity to the Ncam1 3’UTR (2.28-fold increase, *p* < 0.05), whereas concurrent METTL3 knockdown (si-*Mettl3*) completely abolished this interaction, reducing reporter activity to baseline levels (Figure 4G). Consistent with these findings, IGF2BP1 overexpression alone elevated endogenous NCAM1 protein levels by 1.45-fold (*p* < 0.01, Figure 4H). But strikingly, METTL3 depletion not only reversed IGF2BP1-mediated NCAM1 upregulation but also attenuated downstream CREB phosphorylation to control levels (Figure 4I). These coordinated effects establish a mechanistic hierarchy in which METTL3-mediated m6A deposition is essential for IGF2BP1-dependent stabilization of NCAM1 transcripts, thereby modulating CREB signaling output.

### 3.6. Rescue of D-gal-Induced Neuronal Senescence by NCAM1 Restoration Through METTL3-IGF2BP1-m6A-CREB Axis

**Figure 5:**
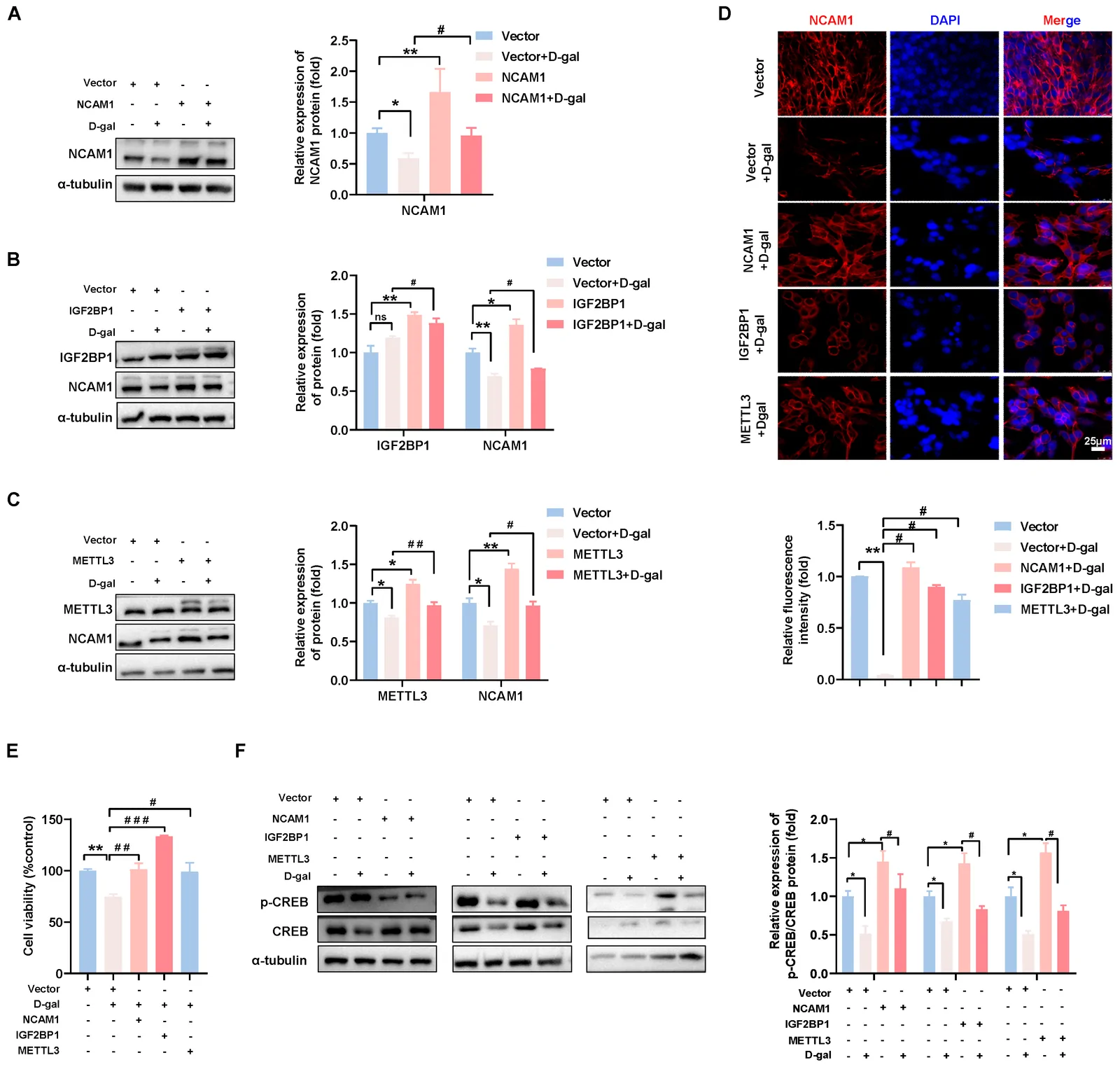
**IGF2BP1/METTL3-mediated m6A-dependent regulation of NCAM1 attenuates D-galactose-induced neuronal senescence via CREB signaling activation:** (**A**–**C**) Western blot analysis was performed to evaluate protein expression in HT22 neuronal cells following 48-h treatment with 200 mM D-gal and NCAM1 (**A**), IGF2BP1 (**B**), METTL3 (**C**) overexpression (n = 3, * compared with the Vector group, # compared with the Vector + D-gal group). (**D**) Immunofluorescence was performed to evaluate NCAM1 expression in HT22 neuronal cells following 48-h treatment with 200 mM D-galactose (D-gal) and NCAM1/IGF2BP1/METTL3 overexpression (n = 3). The lower panel shows the relative fluorescence intensity of each treatment group. (**E**) CCK-8 assays to quantify cell viability of HT22 cells after being treated with 200 mM D-gal for 48 h, followed by individual overexpression of NCAM1, IGF2BP1, and METTL3 through transduction (n = 3). (**F**) Western blot analysis was performed to evaluate CREB and its phosphorylation protein expression in HT22 neuronal cells following 48-h treatment with 200 mM D-gal and NCAM1/IGF2BP1/METTL3 overexpression (n = 3, * *p* < 0.05, ** *p* < 0.01, # *p* < 0.05, ## *p* < 0.01, ### *p* < 0.001).

To investigate whether NCAM1 overexpression could counteract D-galactose-induced cellular senescence, we established an *in vitro* model of accelerated aging by treating HT22 hippocampal neuronal cells with 200 mM D-gal for 48 h. Quantitative immunoblotting analysis revealed a 40% reduction in NCAM1 protein levels after treatment with D-gal (Figure 5A). Notably, overexpression of NCAM1, IGF2BP1, and METTL3 significantly rescued NCAM1 expression, with protein levels reaching 1.7-, 1.4-, and 1.5-fold that of the D-gal group, respectively (Figure 5A–C). Consistent with these findings, immunofluorescence showed that NCAM1 protein abundance was markedly restored in cells overexpressing NCAM1, IGF2BP1, or METTL3 compared with the Vector + D-gal group, and confirmed the membrane localization of NCAM1 (Figure 5D). Parallel assessment of cellular viability demonstrated a 25% reduction in the Vector + D-gal group relative to untreated controls (*p* < 0.01). Importantly, all three overexpression conditions significantly attenuated this deficit, mirroring the trends observed in NCAM1 protein expression (Figure 5E).

To elucidate the downstream signaling consequences of NCAM1 restoration, we examined CREB phosphorylation status across experimental groups. Quantitative immunoblotting revealed a 50% reduction in p-CREB levels in the Vector + D-gal group when compared to untreated controls (*p* < 0.05). Strikingly, individual overexpression of NCAM1, IGF2BP1, and METTL3 not only attenuated this deficit but also significantly enhanced CREB phosphorylation beyond baseline levels (Figure 5F). These findings reveal a direct correlation between m6A-mediated regulation of Ncam1 and activation of CREB signaling, indicating that the anti-senescence effects of IGF2BP1 and METTL3 in neuronal cells are mediated through CREB-dependent transcriptional regulation.

### 3.7. NCAM1 Overexpression Attenuates Age-Related Cognitive Decline in SAMP8 Mice

To systematically evaluate the neuroprotective potential of NCAM1 in age-associated cognitive dysfunction, we engineered a recombinant adeno-associated virus (rAAV) carrying the full-length Ncam1 coding sequence under the control of the CMV promoter (named AAV-Ncam1). Using stereotaxic surgery, the viral particles were bilaterally delivered into the CA1 region of the hippocampus in 5-month-old SAMP8 mice. Behavioral assessments were initiated 4 weeks post-injection to allow sufficient time for transgene expression and functional integration.

**Figure 6:**
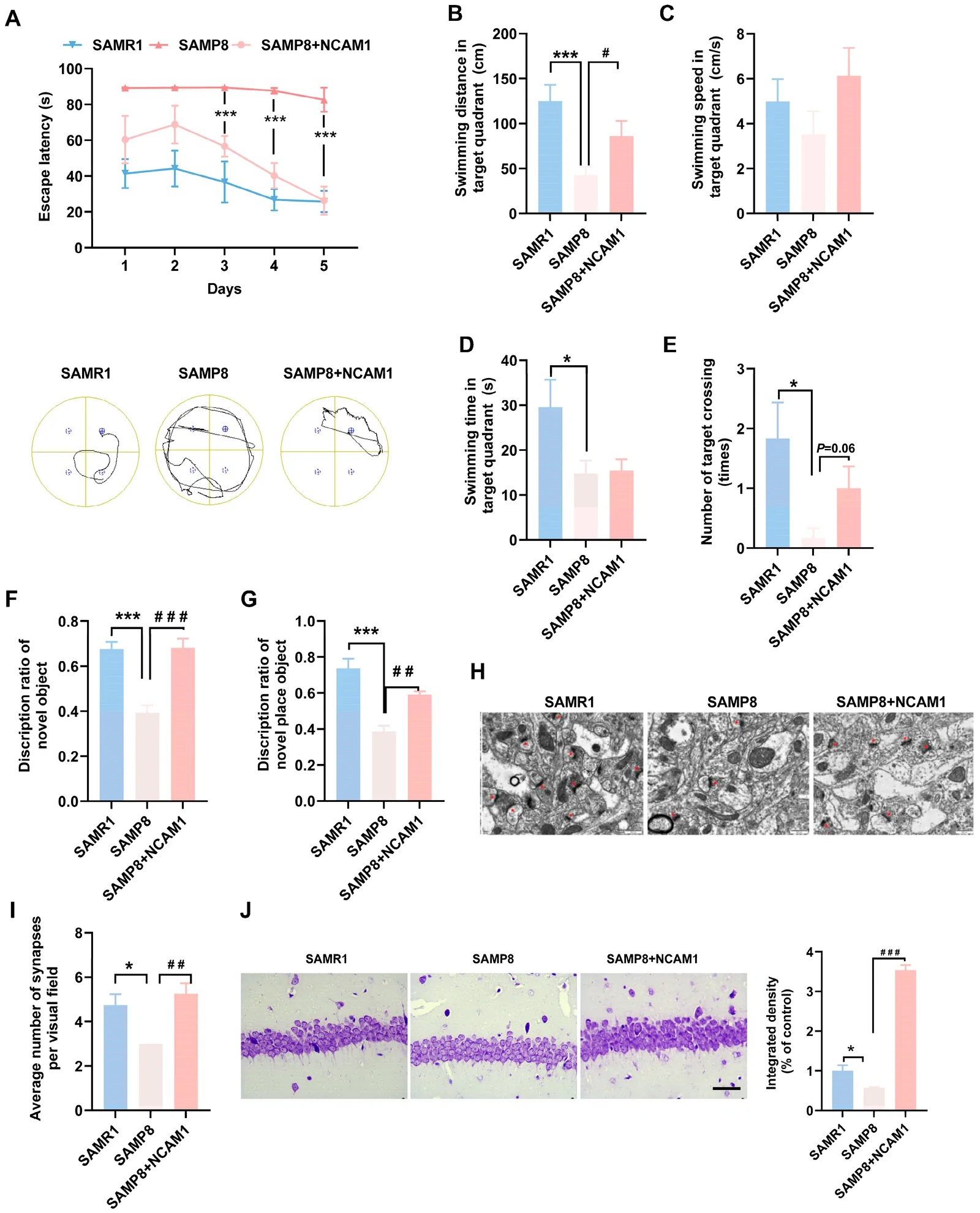
**NCAM1 exerted a restorative impact on learning and memory deficits in aging mice:** (**A**) The escape latency of the MWM experiment during days 1–5 of the learning phase was displayed in the upper panel, and the lower panel showed the representative moving patterns of mice in each group. (**B**) Swimming distance in the target quadrant. (**C**) Swimming speed in the target quadrant during the probe trial. (**D**) Swimming time spent in the target quadrant. (**E**) Number of crossings of the target object quadrant. New object recognition (**F**) and new location recognition test (**G**) in each group (n = 6). (**H**) Classic images of synapses in the hippocampal region. The red stars indicate synapses (Scale bars = 500 nm). (**I**) Quantification of synapse density within the hippocampal regions (n = 4). (**J**) Nissl staining of the CA1 region in the hippocampus of SAMR1, SAMP8 mice and SAMP8 mice overexpressing NCAM1, along with optical density statistical analysis (n = 3, Scale bars = 100 μm) (* *p* < 0.05, *** *p* < 0.001, # *p* < 0.05, ## *p* < 0.01, ### *p* < 0.001).

In the Morris water maze test, NCAM1-overexpressing SAMP8 mice demonstrated significant improvements in spatial learning and memory retention. Quantitative analysis revealed a 37–68% reduction in escape latency in the (SAMP8 + OE-NCAM1) group compared to SAMP8 controls from day 3 to day 5 (*p* < 0.001; Figure 6A, upper panel). Notably, the movement trajectories of NCAM1-overexpressing mice exhibited more efficient and focused search patterns, with their swimming paths concentrated within the target quadrant during probe trials (Figure 6A, lower panel). While swimming speed remained comparable between groups (Figure 6C), the NCAM1-overexpression group showed enhanced spatial memory performance, as evidenced by a 2-fold increase in platform crossing distance (*p* < 0.05; Figure 6B), and extended crossing numbers in the target quadrant (Figure 6E).

The cognitive benefits of Ncam1 overexpression were further validated through comprehensive novel object recognition (NOR) testing. In the standard NOR paradigm, NCAM1-overexpressing mice demonstrated a 1.73-fold increase in novel object exploration time compared to SAMP8 controls (*p* < 0.001; Figure 6F). This enhanced novelty preference was maintained across multiple testing sessions, indicating stable long-term recognition memory. In the spatial variant of the NOR test, where one familiar object was relocated to a novel position, NCAM1-overexpressing mice exhibited a 1.53-fold increase in exploration time for the displaced object (*p* < 0.01; Figure 6G).

We performed detailed ultrastructural analysis of hippocampal synaptic morphology using transmission electron microscopy (TEM). Quantitative assessment revealed a significant decrease in synaptic density in SAMP8 mice compared with SAMR1 controls; in contrast, NCAM1-overexpressing SAMP8 mice exhibited a 75% increase in synaptic density compared to age-matched SAMP8 mice (*p* < 0.01; Figure 6H,I). Nissl staining revealed significant improvements in neuronal health and density in NCAM1-overexpressing mice, with quantitative analysis showing an increased number of Nissl-positive neurons in the hippocampal CA1 region compared to SAMP8 controls (*p* < 0.005; Figure 6J). The cross-sectional area of Nissl-positive neurons was significantly larger in NCAM1-overexpressing mice, indicating enhanced neuronal hypertrophy and improved function. Collectively, these *in vivo* findings demonstrate that NCAM1 overexpression mitigates age-related cognitive decline in SAMP8 mice, potentially through the preservation of synaptic integrity.

### 3.8. IGF2BP1 and METTL3 Overexpression Enhances Cognitive Function in Aged Mice via NCAM1 Upregulation

To systematically investigate the effect of IGF2BP1 and METTL3 in age-related cognitive impairment, we generated recombinant adeno-associated viruses (rAAVs) carrying the coding sequences of Igf2bp1 and Mettl3 under the control of CMV promoters. Using stereotaxic surgery, we bilaterally delivered these viral particles into the hippocampal CA1 region of 5-month-old SAMP8 mice. Behavioral assessments were initiated four weeks post-injection to allow for optimal transgene expression and functional integration.

**Figure 7:**
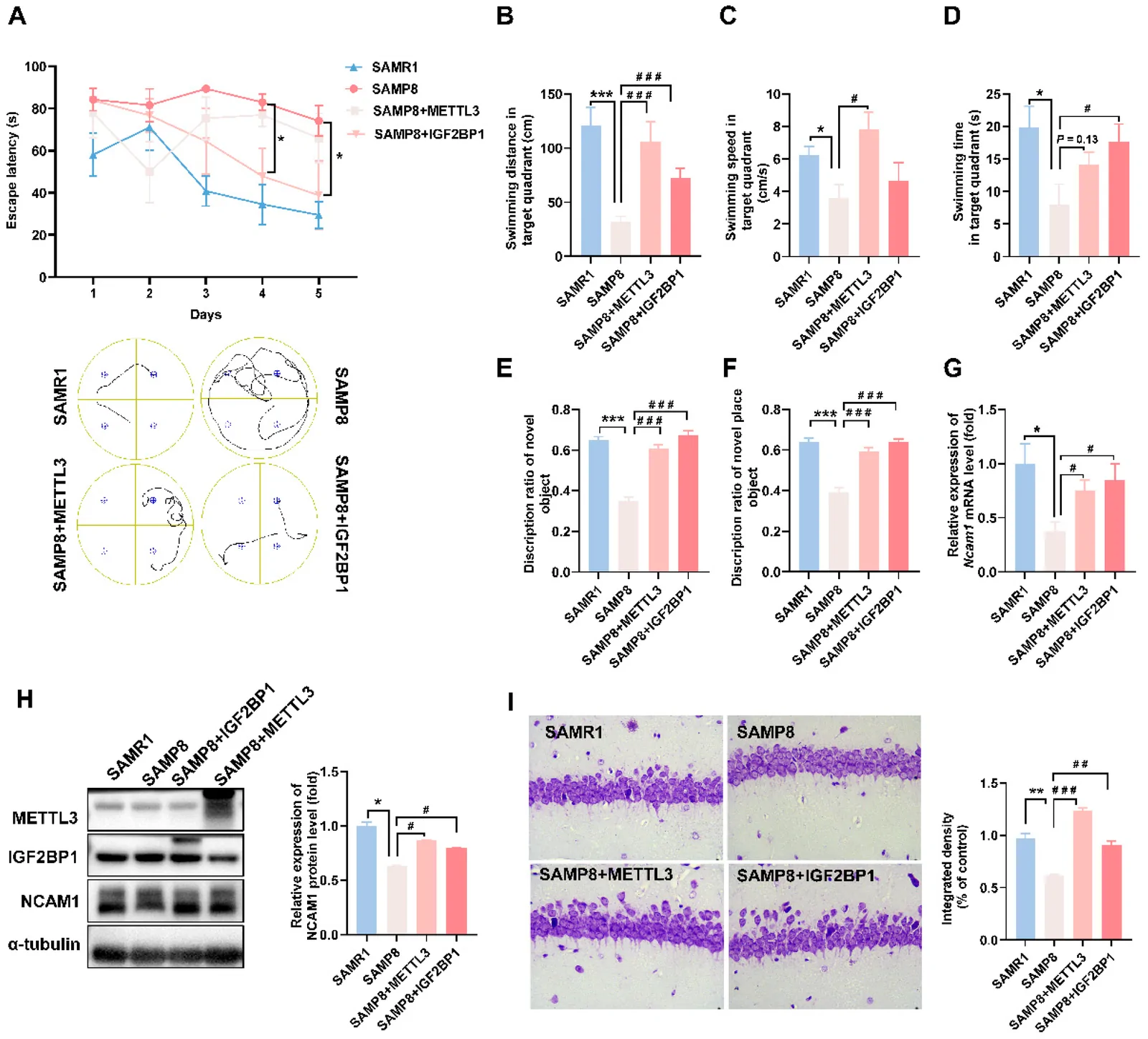
**The rescuable effect of METTL3 and IGF2BP1 on the learning and memory function decline:** (**A**) The escape latency of the Morris water maze experiment during days 1–5 of the learning phase was displayed in the upper panel, and the lower panel showed the representative movement patterns of mice in each group. (**B**) Swimming distance in the target quadrant. (**C**) Swimming speed in the target quadrant during the probe trial. (**D**) Swimming time in the target quadrant. (**E**) New object recognition and (**F**) new location recognition tests in each group (n = 6). (**G**) The hippocampal mRNA expression of *Ncam1* across various mouse cohorts was quantified (n = 6). (**H**) The hippocampal protein expression of NCAM1 across various mouse cohorts was quantified (n = 6). (**I**) Nissl staining of the hippocampus of the SAMR1, SAMP8, SAMP8 + METTL3 and SAMP8 + IGF2BP1 groups and statistical analysis of optical density (n = 3, Scale bars = 100 μm). (* *p* < 0.05, ** *p* < 0.01, *** *p* < 0.001, # *p* < 0.05, ## *p* < 0.01, ### *p* < 0.001).

In the MWM test, both IGF2BP1- and METTL3-overexpressing SAMP8 mice exhibited significant improvements in spatial learning and memory. Quantitative analysis revealed a 40–50% reduction in escape latency in the IGF2BP1-overexpressing groups compared to SAMP8 controls from day 3 to day 5 (*p* < 0.01; Figure 7A, upper panel). The movement trajectories of these mice demonstrated more efficient and focused search patterns, with a higher concentration of swimming paths within the target quadrant during probe trials (Figure 7A, lower panel). METTL3- and IGF2BP1-overexpressing groups showed enhanced spatial memory performance, as evidenced by a 3.3-fold and 2.3-fold increase in swimming distance in the target quadrant (*p* < 0.05; Figure 7B). Intriguingly, both METTL3- and IGF2BP1-overexpressing mice exhibited increased swimming time in the target quadrant compared to SAMP8 mice (*p* = 0.13 for the METTL3 group, *p* < 0.05 for the IGF2BP1 group; Figure 7D).

The cognitive benefits of METTL3 and IGF2BP1 overexpression were further validated through novel object recognition (NOR) testing. In the standard NOR paradigm, METTL3- and IGF2BP1-overexpressing mice demonstrated a 1.75-fold and 1.93-fold increase, respectively, in novel object exploration time compared to SAMP8 controls (*p* < 0.001; Figure 7E). This enhanced preference was consistent across multiple testing sessions, indicating stable long-term recognition memory. In the spatial variant of the NOR test, METTL3- and IGF2BP1-overexpressing mice exhibited a 1.50-fold and 1.60-fold increase compared to SAMP8 mice, respectively, in exploration time for the displaced object (*p* < 0.001; Figure 7F).

To mechanistically link the observed behavioral improvements with molecular changes, we quantified NCAM1 expression at both transcriptional and translational levels following IGF2BP1 and METTL3 overexpression. Quantitative RT-PCR analysis revealed a 2.0-fold increase in NCAM1 mRNA levels in METTL3-overexpressing mice and a 2.2-fold increase in IGF2BP1-overexpressing mice compared to SAMP8 (*p* < 0.05; Figure 7G). Western blot analysis revealed no significant changes in METTL3 and IGF2BP1 protein levels in the SAMP8 group, but showed corresponding increases in NCAM1 protein expression, with IGF2BP1 and METTL3 overexpression elevating NCAM1 levels by 1.4-fold and 1.3-fold, respectively. (*p* < 0.05; Figure 7H). Nissl staining demonstrated improved neuronal health and density in both METTL3- and IGF2BP1-overexpressing mice (*p* < 0.05; Figure 7I). The cross-sectional area of Nissl-positive neurons was also significantly larger in both overexpression groups, indicating enhanced neuronal function.

Collectively, these findings demonstrate that METTL3 and IGF2BP1 overexpression mitigates age-related cognitive decline in SAMP8 mice, potentially through the enhancement of neuronal plasticity.

## 4. Discussion

To elucidate the molecular mechanisms underlying cognitive decline in SAMP8 mice, we performed m6A epitranscriptomic microarray analysis on hippocampal tissues. Our analysis identified 346 differentially expressed m6A-modified transcripts, with 135 hypermethylated and 211 hypomethylated genes in SAMP8 mice compared to SAMR1 controls. Functional annotation revealed significant alterations in neurodevelopmental processes and aging-associated pathways, implicating m6A epitranscriptomic dysregulation in age-related neuronal dysfunction. These findings suggest that m6A modifications play a crucial role in modulating gene expression and maintaining cognitive function in aging brains.

Among the candidate genes identified in our dataset, RELN represents an interesting comparator. RELN is well-established for its predominant roles in early brain development and neuronal migration [32], whereas its expression remained unaltered in the SAMP8 accelerated aging mouse model in our study (Figure 1E). Therefore, we prioritized NCAM1 for further mechanistic validation, given its robust changes in both m6A modification and transcript abundance under aging conditions. NCAM1 is a crucial component of the central nervous system’s extracellular interface, playing a key role in modulating neuron–neuron adhesion, promoting neurite outgrowth, enhancing synaptic plasticity, and facilitating learning and memory [33]. Our luciferase reporter mutagenesis assays demonstrated that each predicted m6A motif within the Ncam1 transcript contributed to its overall m6A-dependent regulation, indicating that these motifs function in a cooperative multi-site manner. This observation is consistent with previous epitranscriptomic work showing that multiple m6A motifs within one transcript can collectively govern mRNA fate, instead of one single dominant site [34].

It should be emphasized that the above mechanistic findings derived from HT22 cell cultures only demonstrate the regulatory role of the METTL3/IGF2BP1-NCAM1 axis in neuronal senescence and aging-associated cellular phenotypes at the cellular level, and cannot directly substantiate effects on cognitive impairment at the organismal level. The functional evidence that this axis ameliorates age-related cognitive decline is independently derived from in vivo behavioral experiments in aged SAMP8 mice, including Morris water maze and novel object recognition assays. The present study provides compelling evidence that IGF2BP1 and METTL3, key regulators of m6A-dependent RNA metabolism, play crucial roles in mitigating age-related cognitive decline through the stabilization and translational regulation of Ncam1. Our findings demonstrate that both IGF2BP1 and METTL3 overexpression significantly improves cognitive performance in senescence-accelerated mice through distinct yet complementary mechanisms. The observed upregulation of Ncam1 following IGF2BP1 and METTL3 overexpression aligns with emerging evidence that m6A modifications serve as critical regulators of synaptic plasticity-related genes [32]. Our data suggest that METTL3-mediated m6A deposition enhances the binding affinity of IGF2BP1 to Ncam1 transcripts, thereby stabilizing the mRNA and promoting its translation. This cooperative regulation is consistent with recent studies demonstrating the synergistic effects of m6A writers and readers in gene expression [35,36]. In conclusion, NCAM1 acts as a critical downstream effector that transduces METTL3-mediated m6A modification and IGF2BP1-dependent mRNA stabilization into functional changes in synaptic plasticity and cognitive decline.

The differential cognitive profiles observed between IGF2BP1- and METTL3-overexpressing mice provide novel insights into their functional specialization. IGF2BP1’s superior effects on spatial learning may stem from its rapid regulation of immediate early genes, which are crucial for initial memory encoding [37]. Conversely, METTL3’s stronger impact on long-term memory retention likely reflects its role in sustaining synaptic protein synthesis, a process essential for memory consolidation [38]. Our ultrastructural and molecular analyses reveal that NCAM1 overexpression increased synaptic density. These findings are particularly significant given the well-documented decline in synaptic plasticity during aging [39,40]. The observed improvements in dendritic complexity and neuronal health further highlight the critical role of the Ncam1 m6A modification process in age-related neurodegenerative disorders.

CREB phosphorylation is crucial for memory formation, and our study revealed a robust positive correlation between NCAM1 expression levels and CREB activation status. SiRNA-mediated NCAM1 knockdown reduced p-CREB levels, while NCAM1 overexpression enhanced CREB phosphorylation. Similarly, depletion of IGF2BP1 or METTL3 reduced p-CREB levels, while their overexpression increased CREB phosphorylation. These results indicate that NCAM1 acts upstream of CREB phosphorylation, a relationship that has been validated by numerous studies [41,42], and further demonstrate that IGF2BP1 and METTL3 modulate CREB signaling via m6A-mediated RNA metabolism.

While our study provides substantial mechanistic insights, several limitations should be acknowledged. First, our functional evidence largely relies on AAV-mediated overexpression within the mouse hippocampus, which drives non-physiologically high protein levels and may not fully recapitulate the moderate endogenous changes in METTL3 and IGF2BP1 that occur during physiological brain aging. The long-term consequences of such overexpression for brain homeostasis also remain to be further explored. Second, although we have uncovered the METTL3-IGF2BP1-NCAM1 regulatory axis in the SAMP8 accelerated-aging mouse model, direct extrapolation of these findings to human brain aging requires caution. Future studies employing human post-mortem brain tissues or human neuronal models are needed to verify whether this regulatory pathway is conserved in humans. Third, as broad-acting global m6A regulators, METTL3 and IGF2BP1 target hundreds of transcripts in addition to NCAM1; their genetic manipulation may therefore induce transcriptome-wide off-target perturbations, which should be carefully considered when interpreting phenotypic results and developing therapeutic strategies.

Future work should focus on cell-type-specific manipulation of the hippocampal METTL3-IGF2BP1-NCAM1 axis and further dissect the potential synergistic effects of combined METTL3 and IGF2BP1 modulation in brain aging and cognitive impairment. The robust cognitive improvements triggered by METTL3 and IGF2BP1 overexpression highlight their promise as therapeutic targets against age-related cognitive decline. Nevertheless, multiple critical hurdles must be overcome before clinical translation. The off-target risks arising from global perturbation of the m6A machinery warrant thorough evaluation [43], and the optimal functional balance between IGF2BP1 and METTL3 activities must be defined to maximize therapeutic benefits while minimizing adverse outcomes.

## 5. Conclusions

In the present study, we systematically explored the epitranscriptomic mechanism underlying age-related cognitive decline by integrating m6A epitranscriptomic microarray profiling, in vitro neuronal senescence models, and in vivo functional validation using SAMP8-accelerated aging mice. Our multi-omics screening identified Ncam1 as a key candidate gene exhibiting coordinated m6A hypomethylation and transcriptional downregulation in the aging hippocampus, which is tightly associated with impaired synaptic plasticity and cognitive deterioration.

Mechanistically, we delineated a precise regulatory axis whereby the m6A writer METTL3 mediates m6A modification on Ncam1 mRNA, and the m6A reader IGF2BP1 specifically recognizes these modified sites to stabilize Ncam1 transcripts. Aging-induced downregulation of METTL3 reduces m6A deposition on Ncam1, destabilizes its mRNA, and decreases NCAM1 protein abundance, ultimately suppressing downstream CREB signaling and exacerbating neuronal senescence and cognitive deficits. In vitro rescue experiments confirmed that overexpression of METTL3, IGF2BP1, or NCAM1 effectively restores NCAM1 expression, rescues D-galactose-induced neuronal senescence, and recovers CREB phosphorylation activity.

Consistent with cellular observations, in vivo AAV-mediated overexpression of NCAM1, METTL3, or IGF2BP1 in the hippocampal CA1 region significantly ameliorates learning and memory impairments in SAMP8 mice. Mechanistically, elevated METTL3/IGF2BP1 signaling restores Ncam1 m6A modification and expression, improves neuronal survival and Nissl body integrity, and rescues aging-induced synaptic loss, thereby enhancing hippocampus-dependent memory consolidation. Notably, METTL3 and IGF2BP1 exert complementary protective effects on spatial learning and memory retention, revealing functional specialization of m6A writers and readers in regulating cognitive aging.

Collectively, our findings demonstrate a critical epitranscriptomic regulatory paradigm: the METTL3/IGF2BP1-m6A-NCAM1-CREB axis safeguards hippocampal neuronal plasticity and cognitive function during aging. This study provides novel mechanistic insights into m6A-dependent brain aging and establishes the METTL3/IGF2BP1-NCAM1 regulatory cascade as a promising target for the intervention of age-related cognitive decline.

## Data Availability

The original contributions presented in this study are included in the article/Supplementary Material. Further inquiries can be directed to the corresponding authors.

## Supplementary Materials

The following supporting information can be downloaded at: https://www.mdpi.com/article/10.3390/cells15181662/s1. Figure S1: SAMP8 mice exhibit diminished spatial learning and memory capabilities relative to SAMR1 group; Figure S2: Transcriptomic analysis of the hippocampal tissue from SAMP8 and SAMR1 mice; Figure S3: Expression analysis of Mettl3, Igf2bp1, Igf2bp3, and Ncam1 in knockdown and overexpression models; Figure S4: The rescuable effect of NCAM1, METTL3 and IGF2BP1 in aged mice; Figure S5: Plasmid maps for AAV constructs and corresponding transgene sequences; Table S1: Sequences of primers and siRNAs used in this study.
