# Supplementary material for: METTL3/IGF2BP1 Axis Orchestrates m6A-Dependent NCAM1 Preservation to Combat Age-Related Cognitive Decline
Source: Cells. 2026 Sep 15;15(18):1662. doi: 10.3390/cells15181662 (PMC13605611; doi:10.3390/cells15181662)
Supplement: Supplementary file 1 [file cells-15-01662-s001.zip › Figure S5 Plasmid maps for AAV constructs and corresponding transgene sequences.pdf]

Plasmid maps for AAV constructs and corresponding transgene sequences

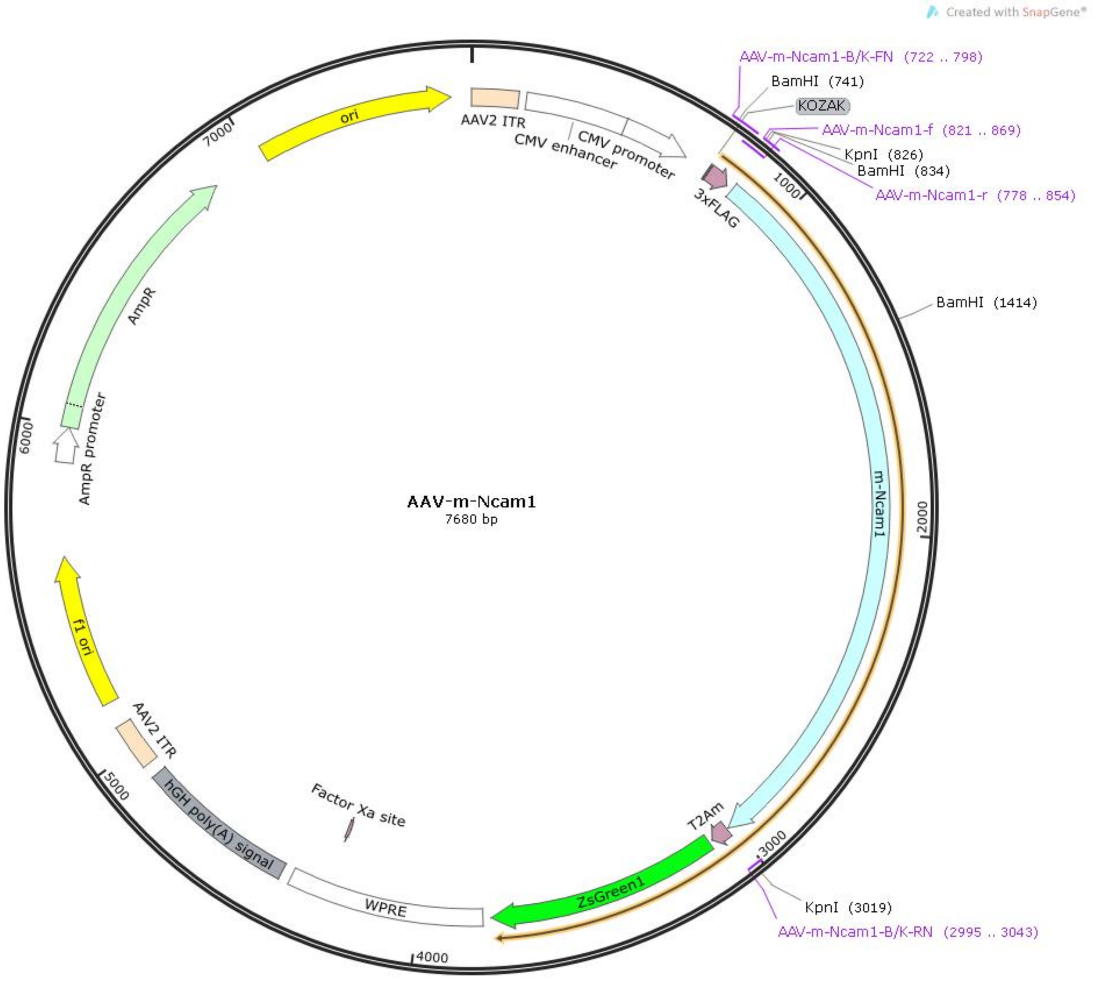

Ncam1 mRNA sequence：

ATGCTGCGAACTAAGGATCTCATCTGGACTTTGTTTTTCCTGGGAACTGCAGTTTCCCT
GCAGGTAGATATTGTTCCCAGCCAAGGAGAAATCAGCGTTGGAGAGTCCAAATTCTTC
CTGTGTCAAGTGGCAGGAGATGCCAAAGATAAGGACATCTCCTGGTTCTCCCCCAATG
GGGAGAAGCTGAGCCCAAACCAGCAGCGGATCTCAGTGGTGTGGAATGATGACGACT
CCTCTACCCTCACCATCTACAACGCCAACATCGACGATGCCGGCATATACAAGTGTGTG
GTCACGGCTGAGGACGGCACCCAGTCTGAGGCCACTGTCAACGTGAAGATCTTCCAG
AAGCTCATGTTCAAGAATGCACCAACCCCACAGGAGTTTAAGGAAGGGGAGGATGCT
GTGATTGTCTGTGATGTGGTCAGCTCCCTGCCTCCAACCATCATCTGGAAACACAAAG
GCCGAGATGTCATTCTGAAAAAAGACGTCCGGTTCATAGTCCTGTCCAACAACTACCT
GCAGATCAGGGGCATCAAGAAAACAGATGAGGGTACTTACCGCTGTGAGGGCAGGAT
CCTGGCCCGCGGGGAAATCAACTTCAAGGACATTCAGGTCATTGTGAATGTACCACCC
ACTGTCCAGGCCAGACAGAGCATCGTGAATGCCACTGCCAACCTGGGCCAGTCTGTCA
CCCTGGTGTGTGATGCCGATGGCTTCCCAGAGCCCACCATGAGCTGGACAAAGGATGG
GGAACCCATAGAGAACGAGGAGGAAGATGACGAGAAGCACATCTTCAGTGATGACAG
CTCCGAGCTGACCATCAGGAATGTGGATAAAAACGACGAGGCCGAATACGTCTGCATC

GCAGAGAACAAGGCTGGCGAGCAGGATGCCTCCATCCACCTCAAGGTCTTTGCAAAG
CCCAAAATCACCTATGTAGAGAATCAGACGGCCATGGAACTAGAGGAGCAAGTCACTC
TGACCTGTGAAGCCTCCGGAGACCCCATCCCCTCCATCACCTGGAGAACATCCACCCG
GAACATCAGCAGTGAAGAAAAGACTCTGGATGGGCACATGGTGGTTCGCAGCCATGC
TCGTGTGTCCTCCTTGACCCTGAAGAGCATCCAGTACACAGATGCTGGAGAATACATCT
GCACTGCCAGCAACACCATCGGCCAGGATTCCCAGTCCATGTACCTCGAAGTTCAATAT
GCTCCCAAGCTCCAGGGCCCTGTGGCTGTGTACACTTGGGAAGGGAACCAGGTGAAC
ATCACCTGTGAGGTCTTCGCCTACCCAAGTGCCACAATCTCCTGGTTCCGAGATGGTCA
GTTGCTGCCAAGCTCCAACTACAGCAATATCAAGATCTACAACACCCCATCTGCTAGCT
ATCTGGAGGTGACCCCTGATTCAGAAAATGACTTTGGAAACTACAACTGTACAGCAGT
GAACCGTATTGGACAGGAGTCCTTGGAATTCATCCTTGTTCAAGCAGACACACCGTCT
TCTCCATCCATTGACCGGGTGGAACCATACTCCAGCACAGCGCAGGTGCAGTTTGATG
AGCCAGAGGCCACAGGGGGCGTGCCCATTCTCAAGTACAAGGCTGAGTGGAAGTCGC
TGGGCGAAGAATCCTGGCATTTCAAGTGGTATGATGCCAAAGAAGCCAACATGGAAGG
CATTGTCACCATCATGGGCCTGAAACCTGAGACGAGGTACTCGGTACGACTGGCGGCC
CTCAACGGCAAGGGGCTGGGCGAGATCAGTGCAGCCACTGAGTTCAAGACACAGCCA
GTCCGGGAACCCAGTGCACCCAAGCTGGAAGGGCAGATGGGAGAGGACGGGAACTC
CATCAAGGTGAACCTGATCAAGCAGGATGACGGAGGCTCCCCCATCAGACACTATCTG
GTCAAGTACAGAGCGCTCGCCTCTGAGTGGAAACCGGAAATCAGGCTCCCATCCGGC
AGTGACCACGTCATGCTCAAGTCCCTGGACTGGAACGCAGAGTATGAAGTCTATGTGG
TAGCTGAAAACCAGCAAGGAAAATCCAAGGCAGCTCACTTTGTGTTCAGGACCTCAG
CCCAGCCCACGGCCATCCCAGCCACCCTGGGCGGAAGCTCCACCTCCTACACCTTGGT
CTCATTGCTTTTCTCTGCGGTGACTCTTCTTCTGCTCTGA

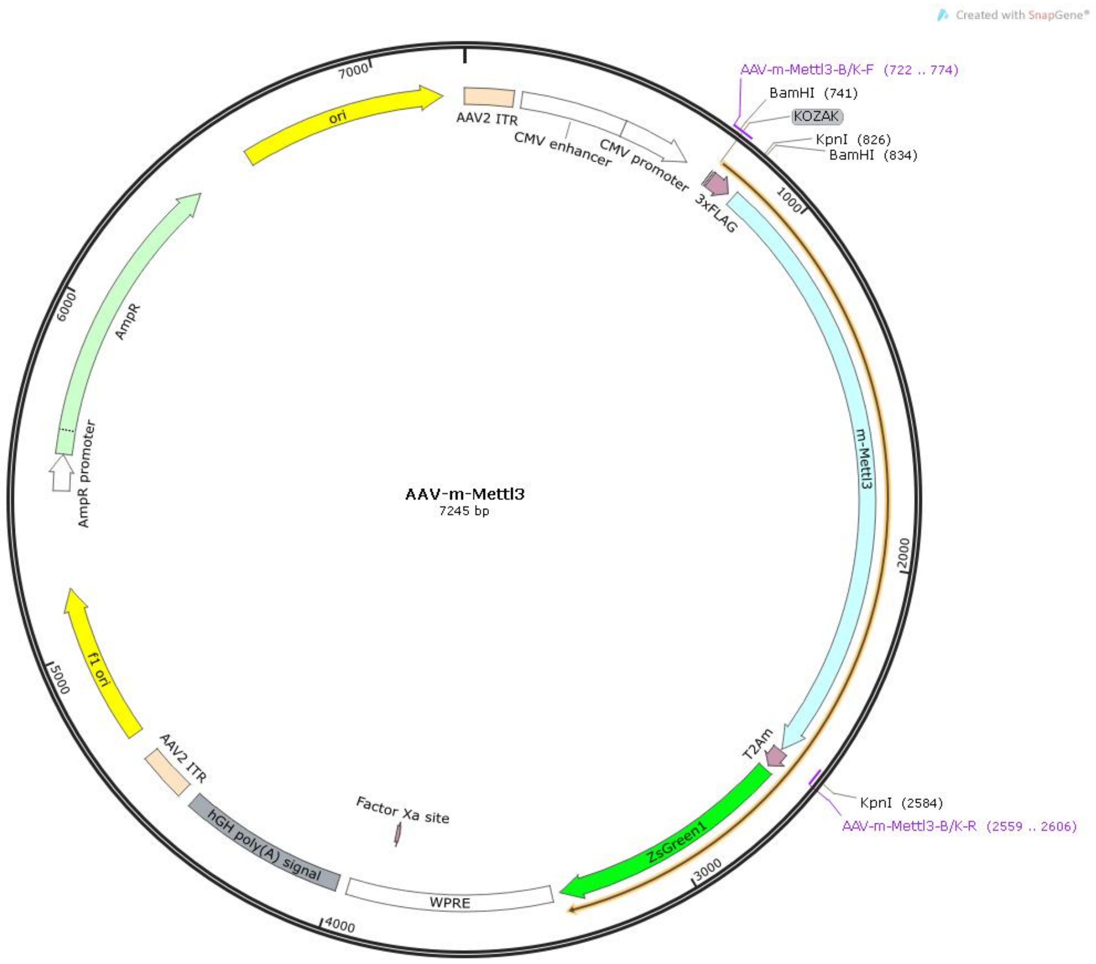

Mettl3 mRNA sequence：

ATGGACTACAAAGACCATGACGGTGATTATAAAGATCATGACATCGACTACAAGGATGA
CGATGACAAGGGTACCGAGCTCGGATCCATGTCGGACACGTGGAGCTCTATCCAGGCC
CATAAGAAACAGCTGGACTCGCTTCGCGAGAGATTGCAGCGGCGACGGAAGCAGGAC
TCTGGGCACTTGGATTTAAGGAACCCAGAAGCGGCACTGTCCCCAACCTTCCGTAGTG
ATAGTCCCGTGCCTACTGCCCCTACCTCTAGCGGCCCTAAGCCCAGCACAACATCTGTG
GCCCCTGAACTAGCTACAGACCCTGAGTTAGAGAAGAAGTTGCTACACCACCTCTCAG
ATCTGGCCTTGACCTTGCCCACTGATGCTGTTTCCATCCGTCTTGCCATCTCTACGCCAG
ATGCACCTGCCACTCAAGATGGGGTAGAAAGCCTTCTCCAGAAATTTGCCGCCCAGGA
GTTGATTGAGGTAAAGCGAGGTCTCCTCCAAGATGATGCACATCCTACTCTTGTAACTT
ACGCTGACCACTCCAAGCTGTCTGCCATGATGGGGGCTGTGGCAGAAAAGAAAGGTC
TTGGAGAGGTAGCAGGGACCATCGCAGGGCAGAAACGGCGTGCAGAACAGGATTTGA
CTACAGTGACCACCTTTGCCAGCTCTTTAGCATCTGGTCTGGCCTCTTCAGCATCAGAA
CCAGCTAAGGAGCCGGCTAAGAAGTCAAGGAAGCACGCTGCCTCCGATGTTGATCTG
GAGATAGAAAGCCTTTTGAACCAACAGTCAACGAAAGAACAGCAGAGCAAGAAGGT
CAGTCAGGAGATCCTAGAGCTATTAAATACCACAACAGCCAAGGAACAGTCCATTGTT
GAAAAGTTTCGCTCTCGAGGTCGGGCCCAGGTGCAAGAATTTTGTGATTATGGGACCA
AGGAAGAGTGCATGAAAGCCAGTGACGCTGACCGGCCTTGTCGCAAGCTGCACTTCA
GACGAATTATCAATAAGCACACTGATGAATCTTTAGGTGACTGCTCTTTCCTTAACACAT
GTTTCCACATGGACACCTGCAAATATGTTCACTATGAAATTGATGCTTGTGTTGATTCTG

AGAGTCCTGGCAGCAAGGAGCATATGCCAAGCCAGGAGCTTGCTCTTACACAGAGTGT
TGGGGGTGACTCCAGTGCTGATCGACTCTTTCCACCTCAGTGGATCTGTTGTGATATCC
GCTACCTGGACGTCAGTATCTTGGGCAAATTTGCAGTTGTGATGGCTGACCCACCTTGG
GATATTCACATGGAGCTACCGTATGGGACATTAACAGATGATGAGATGCGCAGGCTCAA
TATACCAGTGCTACAGGATGACGGCTTTCTTTTCCTCTGGGTCACAGGAAGGGCCATGG
AATTGGGCAGAGAATGTCTGAACCTCTGGGGTTATGAACGGGTGGATGAAATCATCTG
GGTGAAGACTAATCAGCTGCAGCGCATCATTAGGACGGGCCGGACGGGTCACTGGTTA
AACCACGGGAAGGAACACTGCTTGGTTGGTGTTAAGGGAAATCCTCAAGGATTCAAC
CAGGGCCTGGACTGCGATGTGATTGTAGCTGAGGTTCGTTCCACCAGTCATAAACCAG
ATGAAATATATGGCATGATTGAGAGACTGTCCCCTGGCACCCGCAAGATTGAGTTATTT
GGACGACCACACAATGTGCAGCCCAACTGGATTACTCTTGGAAACCAACTGGATGGGA
TACACCTACTAGACCCAGATGTGGTTGCCAGGTTTAAGCAAAGGTATCCGGACGGCAT
CATCTCTAAACCTAAGAATTTATAG

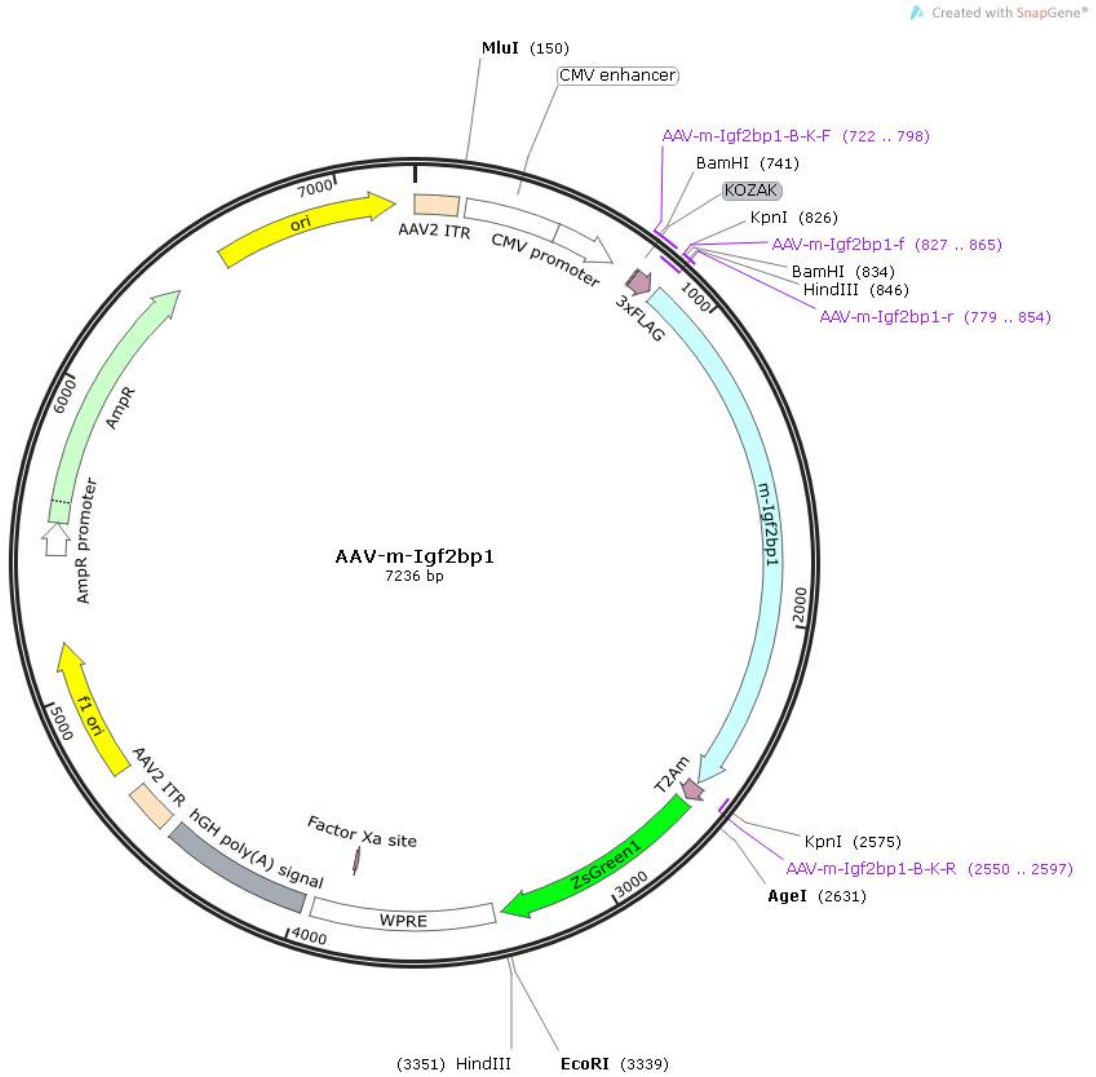

Igf2bp1 mRNA sequence：

ATGAACAAGCTTTACATCGGCAACCTCAACGAGAGTGTGACCCCCGCAGACTTGGAG
AAAGTATTCGCGGAGCACAAGATCTCCTACAGCGGCCAGTTCTTGGTCAAATCCGGCT
ACGCCTTCGTGGATTGCCCCGACGAGCACTGGGCGATGAAGGCCATCGAAACTTTCTC
GGGGAAAGTAGAACTGCAAGGAAAACGTCTAGAGATTGAACACTCAGTCCCCAAAAA
ACAAAGGAGTCGGAAAATACAGATCCGCAATATTCCACCTCAGCTCCGATGGGAAGTG
CTAGATAGCCTGCTGGCTCAGTACGGTACAGTGGAGAACTGTGAGCAAGTGAACACTG
AAAGTGAGACAGCTGTGGTCAACGTCACCTACTCTAACCGGGAGCAGACCAGGCAAG
CTATCATGAAGCTAAATGGCCATCAACTGGAGAACCATGCCCTGAAGGTCTCCTACATA
CCTGATGAGCAGATAACGCAAGGTCCTGAGAATGGGCGTCGTGGAGGCTTTGGGTCTC
GGGGCCAGCCCCGGCAAGGGTCGCCCGTGGCAGCAGGGGCTCCAGCCAAGCAGCAG
CCAGTGGACATCCCTCTCCGGCTCCTGGTGCCTACGCAGTATGTAGGCGCTATCATTGG
CAAGGAGGGTGCCACCATCCGAAACATCACAAAACAGACGCAGTCCAAAATAGACGT
GCATAGGAAGGAGAATGCGGGCGCTGCGGAGAAGGCCATCAGCGTGCATTCAACCCC
TGAAGGCTGCTCCTCCGCGTGCAAGATGATCTTGGAGATTATGCACAAGGAGGCAAAG
GACACCAAAACGGCAGATGAAGTTCCCCTGAAGATCCTGGCTCATAACAACTTCGTCG
GGCGACTCATTGGCAAGGAAGGGCGGAACCTGAAGAAGGTGGAGCAGGACACAGAG

ACGAAGATCACCATCTCATCGCTCCAGGACCTCACGCTCTATAACCCTGAGAGGACCAT
CACTGTGAAGGGCGCCATTGAGAACTGTTGCAGGGCCGAGCAGGAGATCATGAAGAA
AGTTCGAGAGGCTTACGAGAACGACGTGGCCGCCATGAGCTTGCAGTCCCACCTCATC
CCTGGGCTTAACCTGGCTGCTGTAGGTCTCTTCCCAGCTTCATCCAGCGCTGTCCCTCC
TCCTCCCAGCAGTGTCACCGGGGCTGCTCCCTATAGCTCCTTCATGCAGGCTCCGGAGC
AGGAGATGGTACAAGTGTTCATCCCCGCCCAGGCTGTGGGCGCCATCATTGGCAAGAA
GGGCCAGCACATCAAACAACTCTCCCGCTTCGCCAGCGCCTCCATCAAGATTGCACCA
CCAGAAACACCTGACTCCAAAGTTCGAATGGTCGTCATCACTGGACCCCCAGAGGCTC
AGTTCAAGGCCCAGGGAAGAATCTATGGCAAACTAAAAGAAGAGAATTTCTTTGGTCC
CAAGGAGGAAGTAAAGCTAGAGACCCACATACGGGTTCCGGCTTCAGCAGCCGGCCG
TGTCATCGGCAAAGGCGGCAAAACGGTGAATGAGCTGCAGAACTTGACCGCAGCTGA
GGTGGTAGTGCCAAGAGACCAGACCCCGGATGAGAACGACCAAGTCATTGTTAAGAT
CATCGGACATTTCTATGCCAGCCAGATGGCTCAGCGGAAGATCCGAGACATCCTGGCTC
AAGTTAAGCAACAGCACCAGAAGGGACAGAGCAACCTGGCCCAGGCTCGGAGGAAG
TGA
