# Supplementary material for: METTL3/IGF2BP1 Axis Orchestrates m6A-Dependent NCAM1 Preservation to Combat Age-Related Cognitive Decline
Source: Cells. 2026 Sep 15;15(18):1662. doi: 10.3390/cells15181662 (PMC13605611; doi:10.3390/cells15181662)
Supplement: Supplementary file 1 [file cells-15-01662-s001.zip › Supplementary Figure S1-S4.pdf]

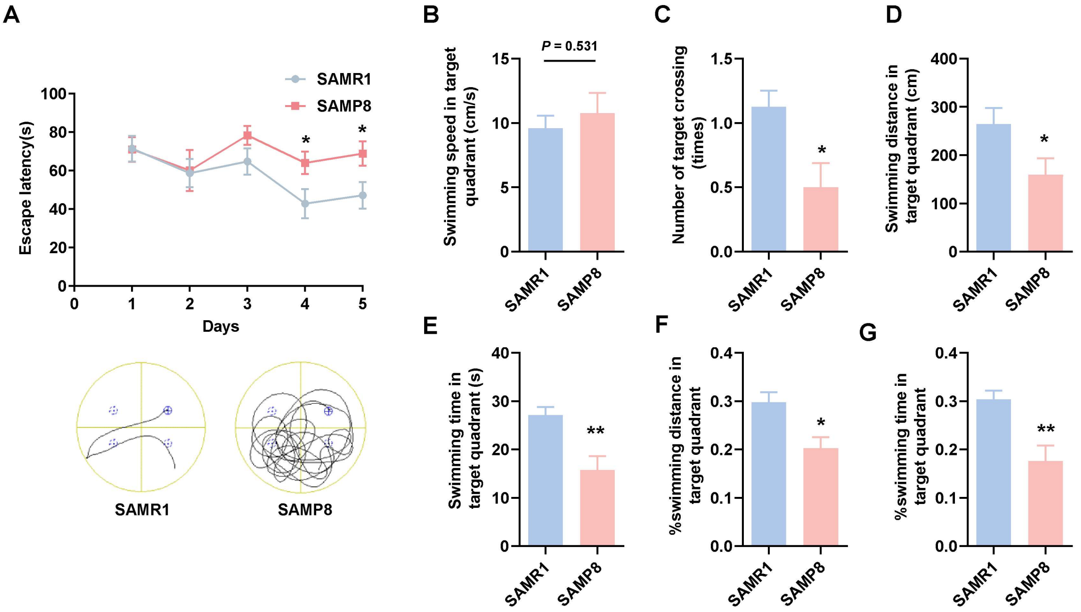

Figure S1. SAMP8 mice exhibit diminished spatial learning and memory capabilities relative to SAMR1 group (A) The escape latency of Morris water maze (MWM) experiment during day 1-5 of learning phase was displayed in the upper panel and the lower panel was the representative moving patterns of mice in each group. (B) Swimming speed in the target quadrant during the probe trial. (C) Number of crossing the target object quadrant. (D) Swimming distance in the target quadrant. (E) Quantification of times spent in the target quadrant. (F) Ratio of swimming distance in the target quadrant. G. Ratio of swimming time spent in the target quadrant. (n = 6 per group, *$P < 0.05$, **$P < 0.01$).

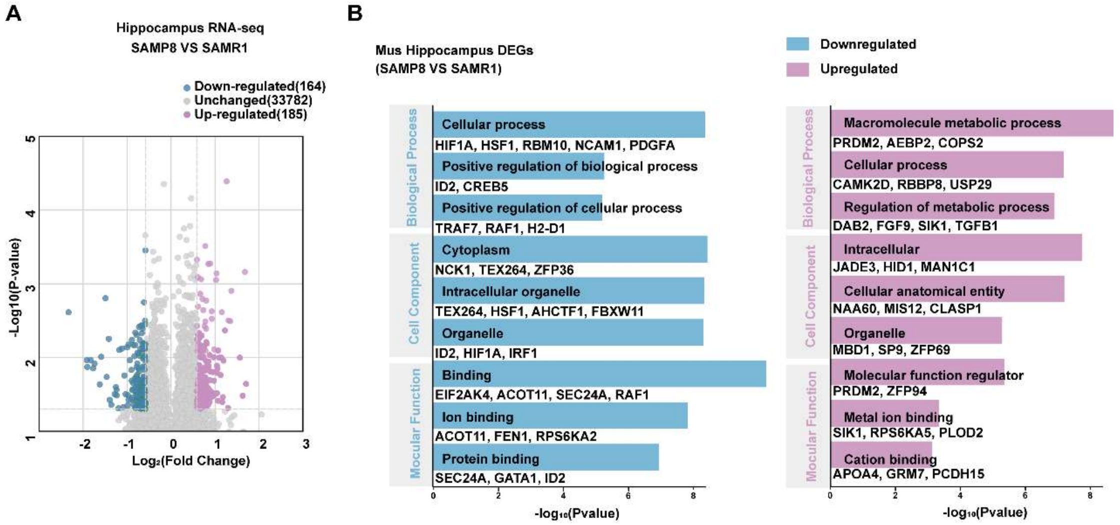

Figure S2. Transcriptomic analysis of the hippocampal tissue from SAMP8 and SAMR1 mice (A) Volcano plots of the different expressed genes between SAMR1 and SAMP8 mice hippocampus. (B) GO enrichment analysis of downregulated and upregulated genes.

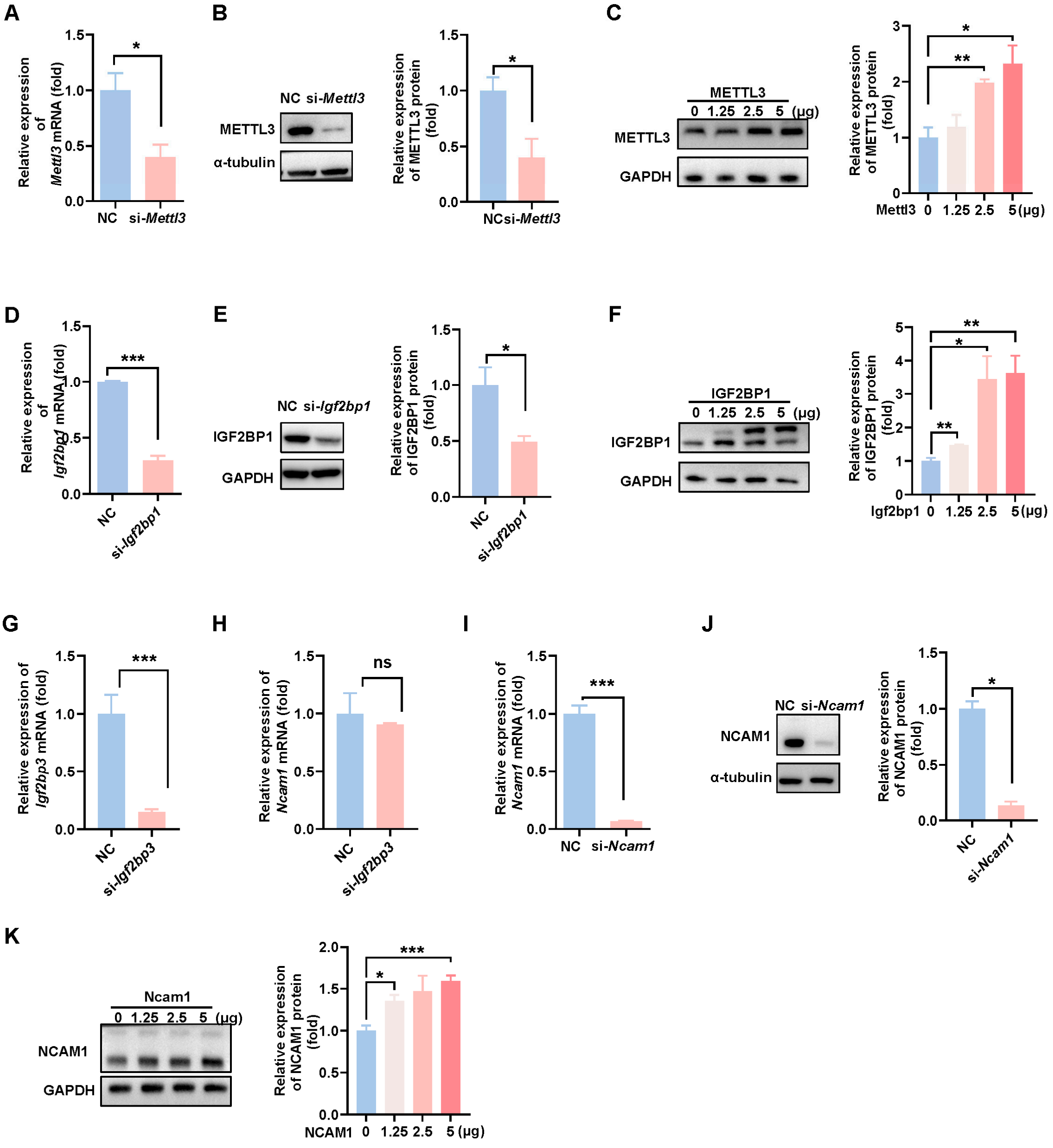

Figure S3. Expression analysis of Mettl3, Igf2bp1, Igf2bp3, and Ncam1 in knockdown and overexpression models (A-B) HT22 cells were transfected with siRNA of *Mettl3*, and mRNA (A) and protein (B) expressions of Mettl3 were analysed respectively. (C) Protein expression of METTL3 after *Mettl3* over expression. (D) mRNA expression of *Igf2bp1* after *Igf2bp1* knockdown. (E) Protein expression of IGF2BP1 after *Igf2bp1* knockdown. (F) Protein expression of IGF2BP1 after *Igf2bp1* over expressed. (G) mRNA expression of *Igf2bp3* after *Igf2bp3* knockdown. (H) Expression level of *Ncam1* mRNA after *Igf2bp3* knockdown. (I) mRNA expression of *Ncam1* after *Ncam1* knockdown). (J) Protein expression of NCAM1 after *Ncam1* knockdown. (K) Protein expression of NCAM1 after *Ncam1* over expressed. (n=3 per group, $*P < 0.05$, $**P < 0.01$, $***P < 0.001$).

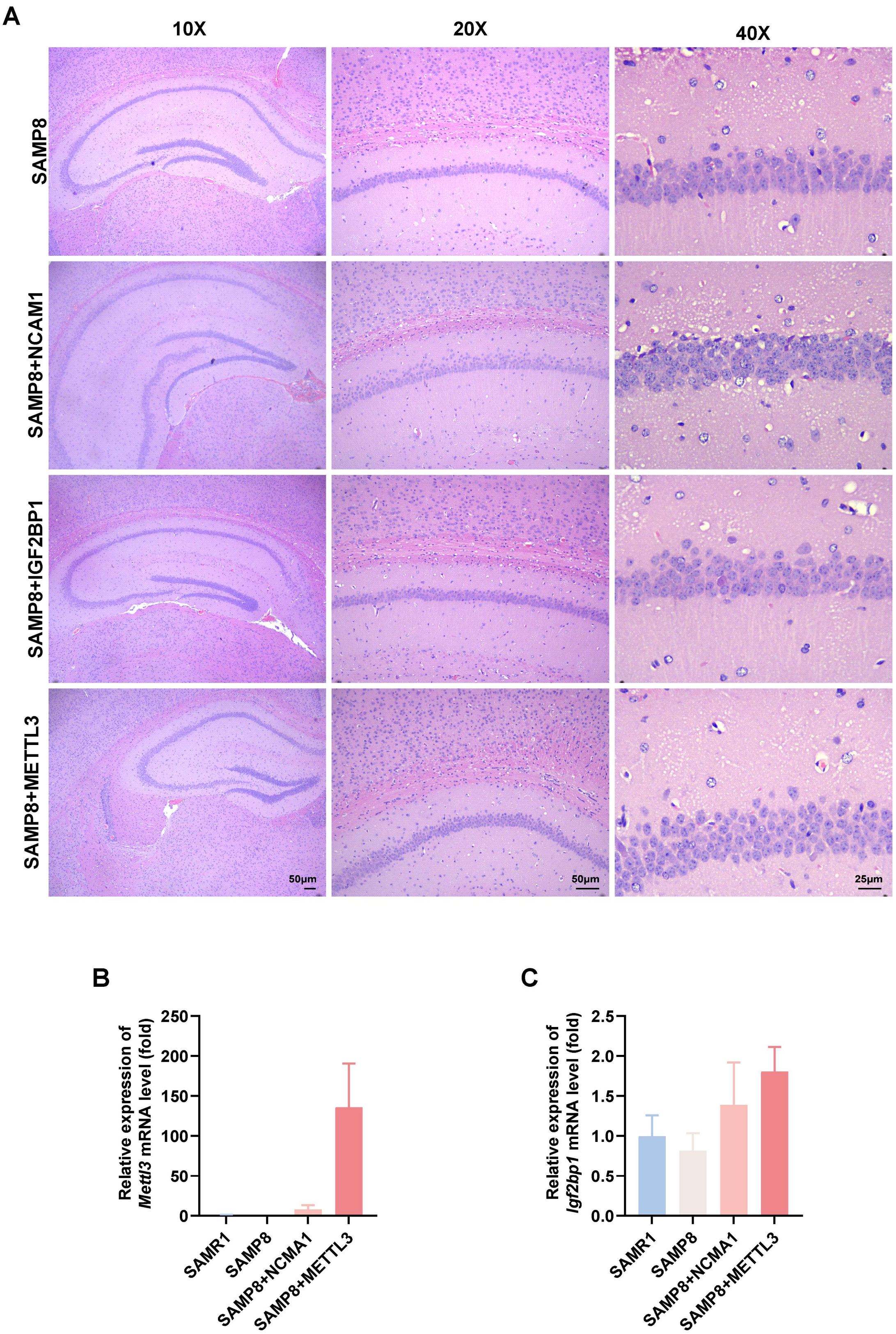

Figure S4. The rescuable effect of NCAM1, METTL3 and IGF2BP1 in aged mice (A) HE staining of hippocampus of SAMP8, SAMP8+NCAM1, SAMP8+METTL3 and SAMP8+IGF2BP1 group (n = 3). (B, C) Relative expression of *Mettl3* and *Igf2bp1* mRNA level of mice hippocampus (n = 6).
